# The bacterial type three secretion system induces mechanoporation of vacuolar membranes

**DOI:** 10.1371/journal.pbio.3003135

**Published:** 2025-05-01

**Authors:** Léa Swistak, Marvin Albert, Camila Valenzuela, Elif Begum Gokerkucuk, François Bontems, Stéphane Tachon, Keith T. Egger, Anastasia D. Gazi, Anna Sartori-Rupp, Cammie F. Lesser, Perrine Paul-Gilloteaux, Jean-Yves Tinevez, Matthijn Vos, Jost Enninga

**Affiliations:** 1 Institut Pasteur, Université Paris Cité, CNRS UMR3691, Dynamics of Host-Pathogen Interactions Unit, Paris, France; 2 Institut Pasteur, Université Paris Cité, Image Analysis Hub, Paris, France; 3 Institut Pasteur, Université Paris Cité, CNRS UMR3569, Structural Virology, Paris, France; 4 Département de Biologie et Chimie Structurales, Institut de Chimie des Substances Naturelles, CNRS UPR2301, Gif-sur-Yvette, France; 5 Institut Pasteur, Université Paris Cité, NanoImaging Core Facility, Paris, France; 6 Institut Pasteur, Université Paris Cité, Ultrastructural BioImaging Core Facility, Paris, France; 7 Center for Bacterial Pathogenesis, Massachusetts General Hospital, Boston, Massachusetts, United States of America; 8 Department of Microbiology, Blavatnik Institute, Harvard Medical School, Boston, Massachusetts, United States of America; 9 Nantes Université, CHU Nantes, CNRS, Inserm, BioCore, US16, SFR Bonamy, Nantes, France; University of Pennsylvania, UNITED STATES OF AMERICA

## Abstract

Endomembrane breaching is a crucial strategy employed by intracellular pathogens enclosed within vacuoles to access the nutrient-rich cytosol for intracellular replication. While bacteria use various mechanisms to compromise host membranes, the specific processes and factors involved are often unknown. *Shigella flexneri*, a major human pathogen, accesses the cytosol relying on the Type Three Secretion System (T3SS) and secreted effectors. Using in-cell correlative light and electron microscopy, we tracked the sequential steps of *Shigella* host cell entry. Moreover, we captured the T3SS, which projects a needle from the bacterial surface, in the process of puncturing holes in the vacuolar membrane. This initial puncture ensures disruption of the vacuole. Together this introduces the concept of mechanoporation via a bacterial secretion system as a crucial process for bacterial pathogen-induced membrane damage.

## Abstract 2

Many intracellular bacteria breach vacuolar membranes to replicate in the host cytosol, but the precise mechanisms remain unclear. This study shows that the human pathogen Shigella flexneri uses its type three secretion system to physically damage endomembranes through mechanoporation to initiate cytosolic access.

## Introduction

Deciphering the strategies of eukaryotic cell invasion by bacterial pathogens is critical to determine how they can be eliminated by the host or by antimicrobial therapies. Intracellular bacteria initially enter eukaryotic cells within vacuoles derived from the plasma membrane. Some pathogens remain vacuole-bound, blocking the trafficking towards lysosomes to avoid degradation and immune detection [1]. Other bacteria breach the vacuolar membrane to access the cytosol where they replicate and spread to neighboring cells [2,3]. Bacterial factors such as pore-forming toxins [4], lipids [5], secretion systems and/or secreted effectors [6–8], have been associated with membrane injury but the exact underlying molecular determinants or mechanisms are often unclear [9]. *Shigella flexneri* (*Shigella*) efficiently escapes its vacuole [10], as broken vacuolar membrane remnants are actively removed from the invading bacteria [11–13]. Cytosolic access by *Shigella* relies on the Type Three Secretion System (T3SS) [14], a specialized molecular apparatus that forms a channel, bridging the bacterial envelopes and the host cell membrane [15]. The T3SS features a basal body anchored within the bacterial envelope and a protruding needle-like structure to reach target cellular membranes for translocon pore insertion. This enables the injection of effectors proteins in one step into the host cell cytosol [15]. Initial vacuole permeabilization has been thought to be achieved via the translocon proteins IpaB/IpaC [16], although such a mechanism has remained puzzling as many non-endomembrane damaging bacteria have T3SSs and homologous translocon complexes [15]. In particular, the dynamics from host cell entry to membrane injury and how T3SS affects vacuole integrity are unknown.

## Results

### *Shigella* T3SS drives multistep cytosolic access

**Fig 1:**
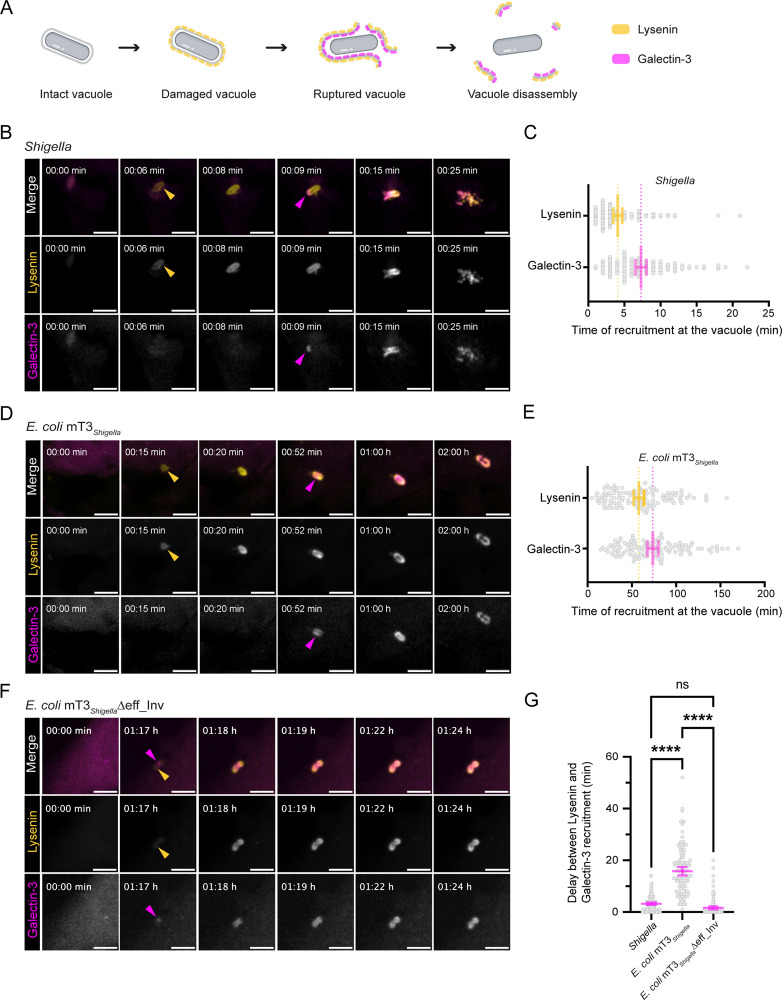
Vacuolar rupture is a multistep process that requires the T3SS for its initiation. **(A)** Graphical illustration of the successive steps leading to *Shigella* cytosolic access monitored using a double reporter cell line: HeLa eGFP-Lysenin mOrange-Galectin-3. Lysenin is recruited at the vacuole upon membrane damage and is followed by membrane rupture, characterized by Galectin-3 recruitment. **(B, D and F)** Representative frames (maximum intensity z-projections) of live-imaging of HeLa cells stably expressing eGFP-Lysenin and mOrange-Galectin-3 infected with *Shigella*, *Escherichia coli* mT3_*Shigella*_ and *E. coli* mT3_*Shigella*_Δeff_Inv (see also S1–S3 Videos). A diffuse (B) or weak (D) signal is visible at time point 0 in both channels, typical for the onset of plasma membrane ruffling or to a slight change in membrane morphology upon bacterial entry (F). Coloured arrowheads indicate the sharp recruitment of Lysenin (yellow) or Galectin-3 (magenta). Scale bars are 5 µm. **(C and E)** Quantification of time of recruitment of Lysenin and Galectin-3 at the *Shigella* (C) or *E. coli* mT3_*Shigella*_ (E) vacuole after entry (time 0). *Shigella*: Lysenin, 4 ± 3 min and Galectin-3, 7 ± 4 min. *E. coli* mT3_*Shigella*_: Lysenin, 58 ± 32 min and Galectin-3, 74 ± 34 min. Bars represent the mean with 95% CI of *n* = 120 of *N* = 3. **(G)** Quantifications of the time delay between the initial recruitment of Lysenin (damage) and Galectin-3 (rupture) at the *Shigella, E. coli* mT3_*Shigella*_ and *E. coli* mT3_*Shigella*_Δeff_Inv vacuoles. Mean delay of 3 ± 3 min for *Shigella*, 16 ± 9 min for *E. coli* mT3_*Shigella*_ and 2 ± 3 min for *E. coli* mT3_*Shigella*_Δeff_Inv. Bars represent the mean with 95% CI, ns = non-significant, ****p < 0.0001, Welch’s *t t*est. *Shigella n* = 120, *E. coli* mT3_*Shigella*_ *n* = 120, *E. coli* mT3_*Shigella*_Δeff_Inv *n* = 120 of *N* = 3. The data underlying Fig 1 can be found in S1 Data.

We aimed to analyze the implication of the T3SS in the early steps of *Shigella* cytosolic access at high spatiotemporal resolution. For this purpose, we established a double reporter HeLa cell line expressing eGFP-Lysenin and mOrange-Galectin-3 to simultaneously track pathogen-induced vacuolar damage, and subsequent vacuolar rupture using time-lapse confocal imaging (Fig 1A). Upon vacuolar damage, sphingomyelins translocate to the cytosolic membrane leaflet, triggering detection by Lysenin [17]. This is followed by irreversible rupture of the vacuolar membrane, exposing glycans to the cytosol and enabling Galectin-3 to diffuse into the vacuolar lumen [18]. This sequence of events precedes later stages of vacuolar disassembly.

We used our dual reporter to investigate the direct role of the *Shigella* T3SS in membrane breaching and elucidate its implication in vacuolar damage or rupture. To this end, we employed engineered *Escherichia coli* strains expressing functional *Shigella* T3SSs [14,19], including the translocon pore proteins IpaB and IpaC (S1 Fig). To bypass the need for specific *Shigella* effectors for host cell entry, we utilized two *E. coli* systems: *E. coli* mT3_*Shigella*_ [19] expressing the structural components of the T3SS plus a reduced set of entry effectors (IpaA, IpgB1, IcsB, IpgD), and *E. coli* mT3_*Shigella*_Δeff_Inv [14] expressing only structural components of the T3SS while lacking entry effectors but exploiting *Yersinia* Invasin (Inv) [20] for receptor-mediated entry.

At 2 and 3 h post-infection respectively, immunofluorescence showed that both *E. coli* mT3_*Shigella*_ and *E. coli* mT3_*Shigella*_Δeff_Inv, invaded our reporter cell line (S2 Fig). Time-lapse microscopy revealed the dynamics of Lysenin and Galectin-3 recruitment at vacuoles containing *Shigella*, *E. coli* mT3_*Shigella*_ and *E. coli* mT3_*Shigella*_Δeff_Inv (Fig 1B, 1D, and 1F). Shortly after epithelial cell entry (average 4 ± 3 min), *Shigella* vacuoles were uniformly Lysenin-positive, indicating membrane damage. The abrupt onset of the Lysenin signal around the entire vacuole is due to the rapid lateral diffusion of sphingomyelins within the cytoplasmic membrane leaflet upon flipping at sites of membrane injury [21,22]. This step was always followed by progressive Galectin-3 recruitment (average 7 ± 4 min after entry, Fig 1C), consistent with the kinetics of Lysenin and Galectin-8 recruitment at the vacuole [17], thereby validating our dual-reporter system. Monitoring *E. coli* mT3_*Shigella*_ cytosolic access, we noted extended delays of Galectin-3 recruitment after the onset of Lysenin signal (Fig 1E). For both, *E. coli* mT3_*Shigella*_ and *E. coli* mT3_*Shigella*_Δeff_Inv infections, the vacuolar membrane remained around the bacteria even after rupture (Fig 1D and 1F), suggesting defects in vacuole disassembly, likely due to the absence of specific effectors involved in membrane unpeeling [12].

By examining the delay between vacuolar damage and rupture through Lysenin and Galectin-3 recruitment, we found that for *Shigella*, Galectin-3 was recruited on average 3 ± 3 min after Lysenin (Fig 1G). In contrast, we observed a 5-fold delay in Galectin-3 recruitment to *E. coli* mT3_*Shigella*_ vacuoles, with rupture being detected on average 16 ± 9 min after Lysenin. These results indicate that cytosolic access by *Shigella* occurs in sequential steps: vacuolar damage followed by rupture, each with distinct kinetics. Notably, *E. coli* mT3_*Shigella*_Δeff_Inv vacuoles ruptured on average only 2 ± 3 min after initial membrane damage, similar to *Shigella*, indicating that receptor-mediated entry through Inv promotes vacuole damage and rupture. Together, our results using engineered *E. coli* strains as a model for invasion using a minimal T3SS, hints towards an important role of the T3SS in initiating membrane injury.

### In-cell analysis of T3SS organization

We reasoned that close interactions between T3SSs and the vacuole would be critical to promote host endomembrane injury. We turned to in-cell cryo-electron tomography (cryo-ET), a technique allowing the visualization of bacterial molecular machineries within infected cells [23]. To follow the involvement of the T3SS in the steps leading to vacuolar rupture, we took advantage of cryo-correlative light and electron cryo-microscopy (cryo-CLEM), imaging *Shigella*-infected HeLa cells expressing our stage-specific double fluorescent reporter to target precise invasion steps. Vitrified cells were first imaged using cryo-fluorescence microscopy (cryo-fLM) to localize fluorescently tagged *Shigella*. Guided by the cryo-fLM information, cells were thinned into lamellae at infection sites using cryo-focused ion beam (cryo-FIB) milling [24]. Then, zones of lamellae with intracellular bacteria were imaged by cryo-ET. To precisely determine the infection stages at both the single-cell and single-bacterium level, we developed a high-throughput correlation procedure (see Materials and methods section). Following data acquisition, all imaging modalities were correlated to provide detailed insight into the invasion stage of individual bacteria (S3A–S3C Fig). This cryo-CLEM approach enabled both consistent data collection of intracellular *Shigella* and allowed for precise assessment of vacuolar integrity surrounding each bacterium. Together, integrating in-cell ultrastructural data and functional fluorescence information was crucial for quantitative image analysis of the successive *Shigella* internalization stages.

**Fig 2:**
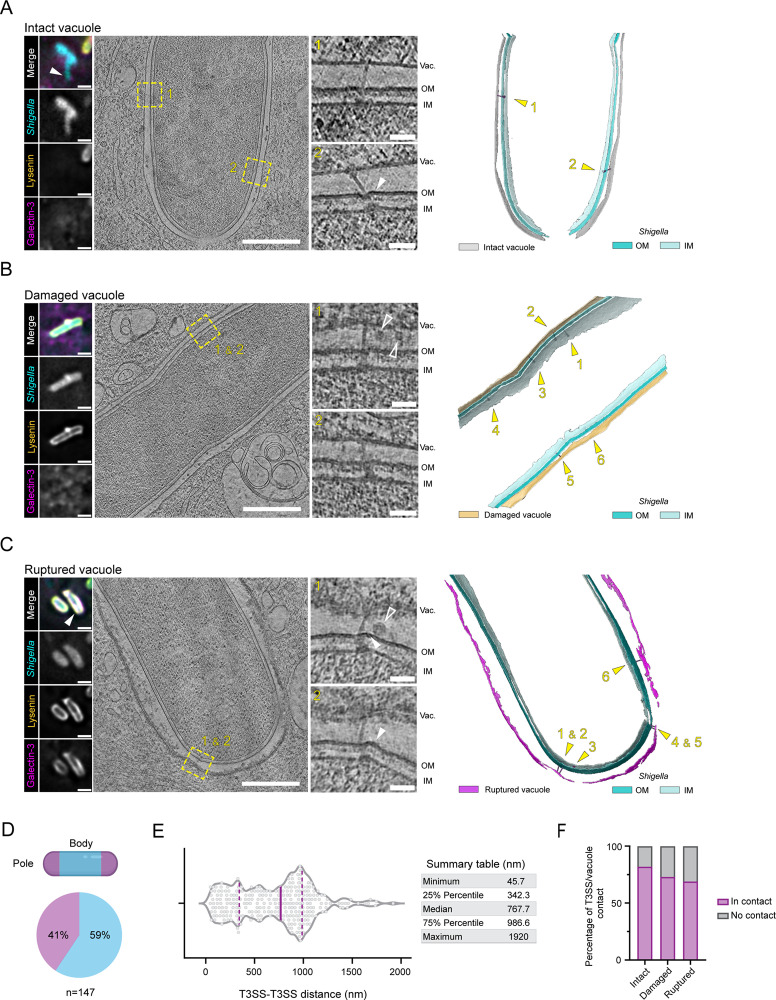
*Shigella* T3SS establishes direct contact with the vacuole membrane during infection. **(A–C)** HeLa eGFP-Lysenin mOrange-Galectin-3 infected with Tag-BFP *Shigella* were processed through a correlative cryo-ET workflow to capture intact (A), damaged (B), or ruptured (C) vacuoles. The first panel displays cryo-fluorescence images of the bacteria imaged by cryo-ET. Scale bars 2 µm. The second panel shows tomographic slices, with T3SSs regions marked by dashed boxes. Scale bars 500 nm. Insets show enlarged views of the T3SSs. Scale bars 50 nm. The right side panel shows membrane segmentation with vacuole coloured according to the infection stage identified by correlative cryo-ET. Yellow arrowheads indicate the T3SSs shown in the insets. White arrowheads point to bacterial membranes deformations while outlined arrowheads point at unknown densities. Vac., Vacuole; OM, Outer membrane; IM, Inner membrane. See also S3A–S3C Fig for full correlation strategy and S4–S6 Videos for tomogram and 3D rendering of vacuole and bacteria membranes. **(D)** Quantification of T3SSs surface distribution (pole/body) across all infection stages. *n* = 147 individual T3SS analyzed. **(E)** Violin plot of the point-based calculation of distances between T3SSs across all infection stages and corresponding summary table. Dashed lines correspond to 25% and 75% percentiles, solid line indicates the median. *n* = 147. **(F)** Contingency graph of the percentage of T3SSs contacting or not the vacuole membrane according to the infection stage. Only the T3SSs for which a basal body and a needle were identified are plotted. Intact *n* = 49, Damaged *n* = 15, Ruptured *n* = 36. The data underlying Fig 2 can be found in S2 Data.

To examine the key stage of *Shigella* cytosolic access, we imaged cells after short infection times (10 min post-infection). At this time point, correlative analysis showed that bacteria were predominantly found entrapped in intact vacuoles, negative for both Lysenin and Galectin-3 (Fig 2A). We also captured the transient stage of vacuole damage, with vacuoles positive for Lysenin but negative for Galectin-3 (Fig 2B). At both stages, the vacuolar membrane tightly surrounded the bacterium, consistent with previously reported volume-imaging of *Shigella* before rupture [11]. Of note, in most of our tomograms, we could spot spherical electron densities within the tight vacuolar lumen possibly resembling small bacterial outer membrane vesicles [25,26] (Fig 2B, insets 1 and 2 outlined arrowheads). We observed the membranes from ruptured vacuoles, positive for both Lysenin and Galectin-3, still in proximity to the invading bacteria but displayed pronounced morphological differences (Fig 2C). Indeed, it was reported that remnants of *Shigella* ruptured vacuole can either disassemble as entire segments, small pieces or fragments, or large sections may remain associated with the bacterium [12,13,27]. In accordance, segmentation of vacuolar membranes from *Shigella* exposed to the cytosol showed significant disruptions including interruptions of varying sizes, delamination, and fragmented membrane remnants (Fig 2C). Notably, damaged and ruptured vacuoles were coated by electron-dense layers that may reflect the accumulation of our fluorescently tagged markers Lysenin and Galectin-3 (S4 Fig).

Throughout the sequential infection stages, we imaged a fair number of densities with the shape and size matching *Shigella* T3SSs (*n* = 122) for which we distinctively identified the major substructures: the bacterial membrane spanning basal bodies and protruding needles. These complexes were exclusively found for T3SS-expressing bacterial strains (S1 and S5 Figs). We also found putative basal bodies without needles (*n* = 25, S6A Fig), suggesting that not all T3SSs are fully assembled prior to bacterial entry. When T3SSs needle complexes and putative basal bodies were identified, they were frequently present in clusters. To gain general insights into the organization of T3SSs at the bacterial surface, we assessed their distribution and found that T3SSs were located to both the poles and the bodies of the bacterial surfaces (41% placed on homogeneously curved membranes and 59% placed on straight membrane segments, respectively; Fig 2D). This observation contrasts with previous reports on the polar secretion of IpaC at host cell invasion [28] and questions a potential redistribution of the *Shigella* T3SSs from a restricted zone to the entire bacterial surface upon host entry, as observed for the T3SSs of *Chlamydia* elementary bodies [29]. To further characterize the localization distribution of T3SSs, we annotated each T3SS, and measured the cartesian distances between nearest neighbors of individual T3SSs. The median T3SS inter-distance was 767.7 nm, but 25% of total T3SSs were only 195 ± 92.5 nm apart (Fig 2E), agreeing with our observation that T3SSs were frequently present in clusters.

T3SSs displayed a range of morphological features in relation to the vacuole membrane, either contacting it or not. Notably, some unengaged T3SSs exhibited a bulb-like density at the needle tip (S3D Fig, Damaged vacuole, inset 5), which we hypothesize to be the needle tip complex, described to be wider than the needle filament, in the case of the *Salmonella* SPI-1 T3SS [30]. Nonetheless, T3SSs were mainly found associated with the vacuole during the early stages of cytosolic access (intact: 82%, damaged: 73%) and remained mostly engaged to vacuolar membrane remnants after vacuolar rupture (rupture: 69%; Fig 2F). These T3SSs displayed significant bending, with the entire structure tilted or with needles deviating from the basal body axis (Fig 2C, insets). Previous work has shown that *Shigella* reroutes the dynein motor complex to generate forces to efficiently remove vacuolar membrane remnants from the bacteria [12]. We speculate that the observed T3SSs distortions may be induced by the forces generated during this process, which would in turn also pull on T3SSs connected to vacuolar remnants. This interpretation was further supported by deformed bacterial membranes observed around tilted T3SSs (Fig 2C, arrowheads). Notably, despite this marked phenotype suggesting the presence of mechanical constraints at the T3SS-vacuole interface, bacteria with distant vacuolar membrane remnants displayed seemingly intact T3SSs at their surface with needles exposed to the host cytosol (S6B Fig). Possibly these T3SSs can be recognized by the inflammasome [31] and may be important for priming *Shigella* secondary infections [32].

### Vacuole tightness constrains the T3SS needle complexes

**Fig 3:**
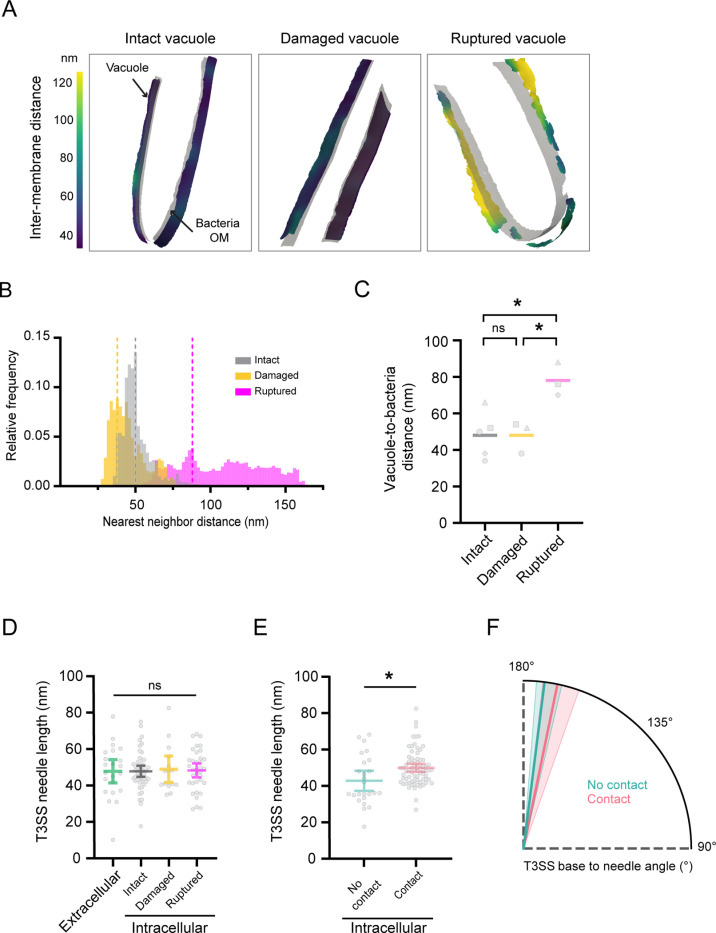
Tight vacuoles with reduced luminal space constrain the T3SS needle complexes. **(A)** Representative membrane surface reconstruction of intact, damaged and ruptured vacuoles coloured by 3D relative distance to the bacteria outer membrane (light gray). Arrows point to relaxed zones. OM: Outer membrane. **(B)** Interleaved histograms of the vacuole-to-bacteria outer membrane nearest neighbor distances of the intact (gray), damaged (yellow) and ruptured (magenta) vacuoles shown in (A). The dashed vertical lines correspond to histogram mode values. **(C)** Comparison of the vacuole-to-bacteria outer membrane distances at different infection stages. Histogram mode values are plotted for intact (*N* = 5), damaged (*N* = 3), and ruptured (*N* = 3) vacuoles. Bars represent the mean (intact: 48.0 ± 12.7 nm, damaged: 48.0 ± 8.7 nm, ruptured: 78.0 ± 9.2 nm), *p < 0.05 and ns = non-significant, one-way ANOVA with Tukey’s multiple comparisons test. **(D)** Quantification of the *Shigella* T3SS needle length along infection stages. Extracellular: *n* = 22 and Intracellular: Intact *n* = 49, Damaged *n* = 15, Ruptured *n* = 39. Bars represent the mean with 95% CI, ns = non-significant, one-way ANOVA with Tukey’s multiple comparisons test. **(E)** Quantification of *Shigella* T3SS needle length depends on whether the needles establish contact with the vacuole or not. Bars represent the mean with 95% CI, *p < 0.05, Welch’s *t* tes*t*. No contact *n* = 25, Contact *n* = 74. **(F)** Quantification of *Shigella* T3SS base to needle angle depends on whether the needles establish contact with the vacuole or not. Median (bold line) and quartile lines (thin lines) are represented. No contact *n* = 25 (25% percentile: 147°, median: 173° and 75% percentile: 176°). Contact *n* = 74 (25% percentile: 161°, median: 168° and 75% percentile: 174°). The data underlying Fig 3 can be found in S3 Data.

Surprisingly, T3SSs with tilted morphologies were also detected at intact vacuoles indicating that the T3SS-vacuole interface is already under substantial tension before membrane rupture. To assess the potential impact of vacuolar constriction on individual T3SSs, we designed an analytical workflow for unbiased and quantitative analysis of intermembrane distances in three dimensions (3D). For this, membranes were semi-automatically segmented and the distances between the vacuole and bacteria outer membranes were determined in a spatially resolved manner using nearest neighbor distance calculation. During early invasion stages, the relative distance between the vacuole and the bacterial outer membrane showed tight spacing (Fig 3A) in line with the skewed distribution of calculated nearest neighbor distances (Fig 3B, gray and yellow). As expected, the space between the bacterium and the ruptured vacuole was enlarged (Fig 3A) with a wider spread distribution of vacuole-to-bacteria outer membrane nearest neighbor distances (Fig 3B, magenta). Vacuolar constriction did not change significantly from intact (48.0 ± 12.7 nm) to damaged vacuoles (48.0 ± 8.7 nm), indicating that membrane injury events occur in localized zones. The increased vacuole membrane to bacteria distances calculated for ruptured vacuoles (78.0 ± 9.2 nm) reflected general alteration of membrane integrity (Fig 3C).

We then measured the needle lengths of T3SSs (Fig 3D) before cell entry (extracellular: 47.8 ± 14.4 nm) and for intracellular bacteria until vacuole rupture (vacuoles intact: 47.8 ± 10.6 nm, damaged: 49.0 ± 13.0 nm, ruptured: 48.3 ± 11.2 nm). The mean T3SS needle length was constant throughout the infection stages and matched the available luminal space before rupture, yet their measured lengths were longer and more heterogeneous than previously reported [33]. Needles that did not establish contact with the vacuolar membrane were significantly shorter, averaging 42.8 ± 13.4 nm length. Notably, T3SS needles in contact with the host membrane averaged 50.0 ± 9.6 nm, with some extending over 50 nm, exceeding the mean gap between the bacteria and the vacuole (Fig 3E). While T3SSs that did not contact the host vacuole were rather straight (Fig 3F; average base to needle angle of 170.4° ± 7.8°), needles in contact with the vacuole showed pronounced bending with regards to the T3SS base (average 165.5° ± 11.8°). Together, this suggests that T3SSs mediate contacts between bacteria and the vacuole and that the vacuolar tightness exerts substantial tension on T3SS needles.

### T3SS-induced mechanoporation of the vacuole

At the early infection stages (Galectin-3 negative) the vacuolar membrane did not exhibit zones that were prominently perturbed. Therefore, we reasoned that early membrane injury events should be assessed at the sites where individual T3SS are in contact with the vacuole. We closely examined the T3SS-vacuole interface prior to vacuolar rupture and correlated the needle length of individual T3SS to the surrounding local vacuole-to-bacteria distances and membrane curvedness [34] as quantitative measure of local membrane deformation in local neighborhood of the T3SS tips. T3SSs not contacting the vacuole had either short needles or were located to zones with relaxed vacuolar membranes (S7A and S7B Fig), indicating that contact requires matching between both the needle length and the inter-membrane space. This interpretation is further supported by the T3SSs with needles lengths fitting the luminal space that showed limited to no constraints on the vacuole or bacterial membranes (S7C Fig). Together, these observations imply an interplay between the needle length and the local vacuolar tightness exerting opposing forces at the T3SS-vacuole interface.

**Fig 4:**
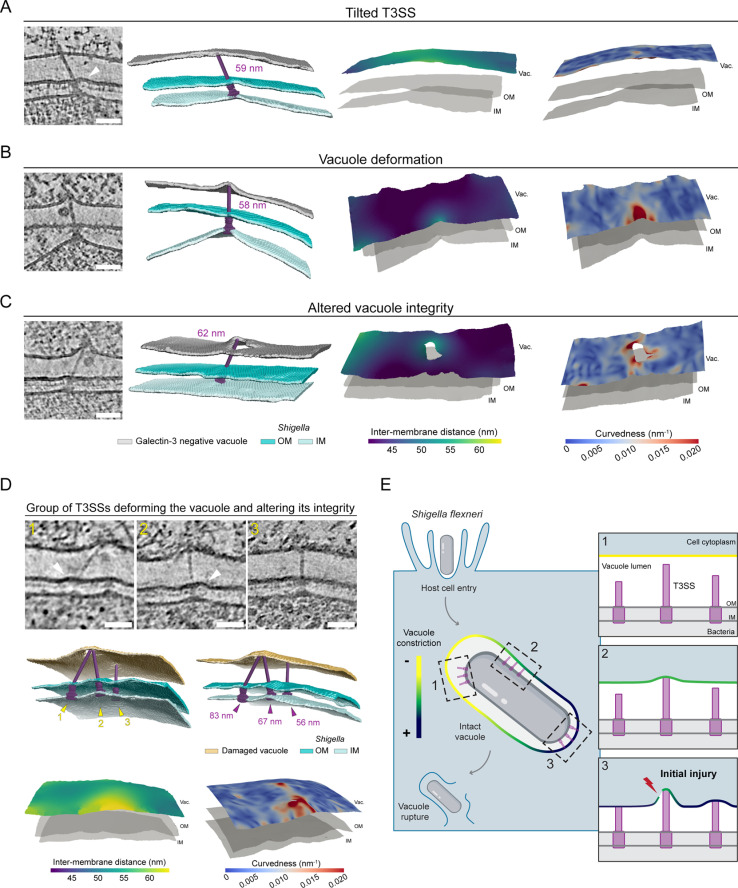
*Shigella* T3SS mechanoporation of constricted vacuolar membranes. **(A–D)** Tomogram slices of the T3SS-vacuole zones before *Shigella* membrane rupture. White arrowheads point to bacterial membranes deformations. Scale bars 50 nm. Corresponding 3D rendering of vacuole and bacteria membranes with *Shigella* T3SS 3D map (EMD-15700) fitted. Galectin-3 negative vacuole (gray), Lysenin-positive damaged vacuoles (yellow), bacteria OM (dark blue), and IM (light blue). The last panels show representative membrane surface reconstruction of the vacuole membrane coloured by 3D distance to the bacteria outer membrane (left) or vacuole membrane curvedness (right). Bacteria membranes are shown in light gray. Vac.: Vacuole, OM: Outer membrane, IM: Inner membrane. See also S8A–S8C Fig for correlations and T3SS positioning information and associated S7–S9 Videos. The data underlying Fig 4 can be found at 10.5281/zenodo.15065516. (A) T3SS with needle length (59 nm) exceeding local luminal space (~50 nm) adopting a tilted insertion within bacteria membranes with OM showing deformation. The vacuole membrane is only slightly deformed at the T3SS contact site. (B) Straight T3SS with needle length (58 nm) exceeding local luminal space (~45 nm), pushing the vacuole membrane forming a steep bulge at T3SS-vacuole contact site. (C) T3SS with needle length (62 nm) exceeding local luminal space (~45 nm) deforming the vacuole membrane up to altering its integrity by physically puncturing it. See also S10 Video. (D) Group of three T3SS localizing in the same zone of the bacteria surface. T3SS n°1 and 2 contact, deform, and perforate the vacuole membrane at the same spot. T3SS n°1 needle (83 nm) is long and seems to deviate from basal body axis showing that strong constraints between T3SS and the vacuole at are play. The locally relaxed vacuole to bacteria space (~50–55 nm) potentially limits additional damage by the T3SS n°3 needle (56 nm) that only slightly deforms the membrane. **(E)** Model of initial vacuolar membrane breaching by T3SS-induced mechanoporation. *Shigella* actively enters host cells in a specialized endomembrane compartment, the vacuole, that is rapidly injured and ruptured for cytosolic access. After host cell entry, the vacuole membrane is intact and tightly juxtaposed to the surface of *Shigella* permitting contact between T3SSs and the endomembrane. In our proposed model, vacuole membrane injury is supported both by the vacuole tightness and the length of individual T3SSs. Assessment of the local T3SS environment showed that T3SSs needles not contacting the vacuole were short or were in zones with a relaxed vacuolar membrane (1: yellow vacuole membrane). With increased vacuolar constriction (2: green vacuole membrane) or longer needles, T3SSs established contact with the endomembrane, underlining the interplay between both, needle length and vacuole tightness, in mediating bacteria/vacuole interaction. Strong vacuolar constriction (3: dark blue vacuole membrane) at sites with longer T3SS needles results in membrane injury resembling mechanoporation.

We next focused on T3SSs with longer needles that did not fit into the tight vacuoles. T3SSs with bent insertions across bacterial membranes (Figs 4A and S7D), only slightly deformed the vacuolar membrane. This indicates that the local vacuole constriction exerts a strong mechanical force on T3SSs with long needles, pushing the T3SS sideways resulting in a tilted complex. At the bacterial level, the T3SS tilt was echoed by deformations of the membranes surrounding the basal body (Figs 4A and S7D white arrowheads). In these cases, the local vacuole tightness is the main factor constricting the T3SSs. In other cases, T3SSs with long needles were inserted straight across the bacterial envelope and deformed the vacuolar membrane locally forming bulges, shown by strong increase of the curvedness at the T3SS-vacuole contact site (Fig 4B). Such strong membrane curvedness was not detected away from the T3SS (S7E and S7F Fig), highlighting the reciprocity between needle length and vacuole constriction leading to local membrane deformation. Further examination of the vacuolar membrane morphologies at these zones revealed membrane lesions very close to the longer needle contact sites (Fig 4C and 4D). These T3SSs were also bent, emphasizing the tensions generated by opposing forces necessary to induce membrane injuries. By segmenting the bacterial and host membranes and docking an available atomic model for the T3SS into our tomogram density we could visualize that the T3SS needle physically injured the vacuole membrane. We observed large holes localized near the long T3SS needles (Fig 4C and S10 Video) and punctures (Fig 4D). We could also spot multiple T3SSs contacting and inducing curvature of the vacuolar membrane in the same area (Fig 4D). Even though these membrane injuries were observed, these vacuoles were still negative for the rupture marker Galectin-3 (S8 Fig). Thus, we propose that the *Shigella* T3SS needle complex initiates vacuole membrane injury by physically deforming it and puncturing a hole, akin to mechanoporation [35].

## Discussion

We previously described *Shigella* vacuolar escape [12], but the mechanism of initial vacuolar membrane injury has remained unclear. Here, we address this knowledge gap showing that the T3SS breaches the vacuolar membrane during the initial phase of cytosolic access. We developed correlative in-cell cryo-ET strategies followed by quantitative analysis of endomembrane morphology to image the transient steps preceding *Shigella* cytosolic access. We propose a new model where membrane damage is induced locally at sites with both constricted membranes and T3SSs with needles exceeding the available vacuolar luminal space (Fig 4E).

Our results showing that endomembrane injury occurs at local T3SS-vacuole contact sites are in line with the detection of *Yersinia* phagosome rupture following T3SS translocon insertions imaged by advanced optical imaging approaches [36]. Translocon complexes form at the point of contact between the T3SS needle tip and host cell membranes [37]. They have been postulated to function similarly to pore-forming toxins, inducing membrane destabilization and red blood cell hemolysis [16,38,39]. However, whether translocon insertion damages membranes remain unclear in the context of host–pathogen interaction. Of note, we never observed recruitment of the membrane damage marker Lysenin to *Shigella* entry foci despite translocon insertion being required for effector secretion and host cell entry. Nevertheless, our detection methods do not exclude a potential role of IpaB and IpaC, as part of the translocon or their secreted fraction, in membrane damage.

Cryo-tomography provided an appropriate resolution to identify zones of vacuole destabilization. Specifically, endomembrane injury could be visualized where the vacuole constricts around long T3SSs needles. In the case of *Yersinia*, needle length regulation has been studied in detail and described to depend on a T3SS protein acting as a molecular ruler [40,41]. In agreement with our observations of *Shigella* T3SS needles having disparate sizes (Fig 3D), needle length regulation in *Shigella* appears to be less stringent [33]. Some T3SS needles in contact with the vacuole slightly deformed it but had bent insertions across the bacterial envelopes (Figs 4A and S7D). This observation supports a model in which the vacuole exerts mechanical forces on the T3SS. The spatial constraints push the T3SS needles leading to deformation of the bacterial membranes around the basal body. We propose that long needles, with a length exceeding the available space between the bacteria and the vacuole, either push the endomembrane or are constrained by the tensions applied from the cytoplasmic zone on the vacuole resulting in the tilting of the T3SS complex. This implies the T3SS-vacuole interface is under major tension. Collectively, our data indicate that initial vacuolar injury arises from the opposing forces exerted by T3SS needles pushing against the endomembrane, combined with the vacuole constricting around the T3SSs. This interplay ultimately leads to vacuolar rupture, implying that vacuole injury is primarily a mechanical process.

A possible explanation of the molecular basis of endomembrane damage would be the increased stiffness [42] of cholesterol-rich nanodomains where *Shigella* T3SSs contact host membranes [43]. Moreover, the *Shigella* vacuole is enclosed in a thick actin cocoon [13] a structure that possibly increases endomembrane resistance to the deformations induced by the T3SS needles. Of note, an actin network often surrounded *Shigella* vacuolar membranes in our datasets (Fig 2C). In addition, *Shigella* are enclosed in very tight vacuoles (Fig 3A–3C) with limited lipid availability which could make the *Shigella* vacuoles prone to T3SS-induced mechanoporation. *Yersinia* has also been described to deform the phagosome membrane at the interface with the T3SS however injuries were not observed [44]. In line with this, other T3SS-bearing pathogens with canonically larger vacuoles do breach their endomembranes [45,46]. *Chlamydia* induces the formation of an actin mesh and encodes for a T3SS but does not rupture its vacuole. As *Chlamydia* vacuoles are larger it is unlikely that its T3SSs contact the vacuolar membrane at a tense interface as observed for *Shigella*. In addition, actin cages around *Chlamydia* vacuoles are different in composition and organization compared to the *Shigella* actin cocoon [47].

We argue that *Shigella* is highly efficient exerting T3SS-induced mechanoporation, but this process may also play a role for other T3SS-bearing pathogens, such as *Salmonella*. Accordingly, there is a correlation between the shrinking of the *Salmonella*-containing vacuole [48,49] and endomembrane breaching [50]. Other pathogens could damage their vacuole through T3SS-mediated mechanoporation, but might have evolved additional mechanisms to efficiently hijack host membrane repair pathways, preventing vacuole rupture and cytosolic escape [51,52]. During, *Shigella* invasion, the tight vacuole, limited lipid availability, and the surrounding actin cytoskeleton might prevent spontaneous resealing of T3SS-induced holes leading to catastrophic membrane injury [53].

The concept of mechanoporation may also take place during endomembrane damage through other secretion systems such as a *Burkholderia thailandensis* Type 6 Secretion System recently shown to be involved in secondary vacuole lysis [54].

Together, we uncover that *Shigella* initial vacuole injury occurs through T3SS-mechanoporation. This establishes a new paradigm in membrane damage by pathogens with a direct involvement of bacterial secretion system during early membrane injury.

## Materials and methods

### Cell culture and stable cell line generation

All HeLa epithelial cell lines used in this study were cultured in Dulbecco’s modified Eagle’s medium (DMEM, Gibco) supplemented with 10% (v/v) heat-inactivated fetal bovine serum (Sigma-Aldrich) at 37 °C, 5% CO_2_ and were continuously monitored for mycoplasma.

The double expressing mOrange-Galectin-3 and eGFP-Lysenin stable cell line was generated using the Sleeping Beauty (SB) transposon system [55]. Briefly, N-terminally eGFP tagged Lysenin was PCR amplified from M6P-GFP-LyseninW20A plasmid [17] (kindly provided by Felix Randow) using the primers Forward_eGFP (5′aggcctctgaggccaccatggtgagcaagggcgag 3′) and Reverse_Lysenin (5′ aggcctgacaggcctcagcccacgacttccagga 3′). The obtained amplicon was cloned into the SB transposon (pSBbi-BLA, addgene plasmid #60526) using the Sfil sites. The resulting pSBbi eGFP-LyseninW20A plasmid was confirmed by sequencing and co-transfected with the vector encoding the SB100X transposase, pCMV(CAT)T7-SB100X (Addgene plasmid #34879) in the monoclonal HeLa pSBbi mOrange Galectin-3 [13] (puromycin resistant). Forty-eight hours post-transfection, cells were switched to selection media containing 10 µg/mL blasticidin (Gibco) and 1.7 µg/mL puromycin (Gibco). After 10 days of culturing in selection media, cells were sorted to select double fluorescence positive clones (eGFP-Lysenin and mOrange-Galectin-3) using a BD FACSAria III Cell Sorter (BD Biosciences). A polyclonal subpopulation with high expression of eGFP-Lysenin was further selected for expansion.

### Bacterial strains and infection for fluorescence microscopy

*Escherichia coli* DH10β (Thermo Scientific), derivative minimal T3SS (mT3) strains: *E. coli* mT3 (pLLX13 *ipaJ* thru *spa40*, TET^R^, KAN^R^ + pNG162-VirB, SPEC^R^) [19] and *E. coli* mT3_*Shigella*_Δeff_Inv (pLLX13 *ipaJ* thru *spa40ΔipaA,* Δ*ipgB1*, Δ*icsB*, Δ*ipgD*, TET^R^, KAN^R^ + pNG162-VirB, SPEC^R^ + pRI203, CM^R^, AMP^R^) [14] were grown in lysogeny broth (LB) at 37 °C with shaking. For the mT3 strains, LB was supplemented according to strain specification with 20 µg/mL tetracycline, 50 µg/mL kanamycin, 100 µg/mL ampicillin, and 100 µg/mL spectinomycin (all from Sigma-Aldrich). For *E. coli* infection experiments, bacteria were diluted in LB at 1:50 from an overnight culture and grown at 37 °C. In the case of the mT3 strains, T3SS expression was induced with 1mM IPTG (Thermo Scientific). At an optical density of 600 nm (OD_600_) ~0.45 bacteria were harvested by spinning and washed twice in EM buffer (120 mM NaCl, 7 mM KCl, 1.8 mM CaCl_2_, 0.8 mM MgCl_2_, 5 mM glucose, 25 mM HEPES, pH 7.3). Bacteria were diluted to a multiplicity of infection (MOI) of 100 in EM buffer and spun down on the cells for 10 min at 180*g*, at room temperature (RT).

*Shigella* strains were derivatives of the WT *Shigella flexneri* M90T, when indicated they carried the pGG2-eGFP or pGG2-TagBFP [56] plasmids, for constitutive expression of eGFP or TagBFP, respectively. *Shigella flexneri* BS176, a M90T derivative lacking the virulence plasmid, was used as control for secretion assays [57]. *Shigella* strains were plated on trypticase soy (TCS) agar supplemented with 0.01% Congo Red (Sigma-Aldrich) and liquid cultures were grown in TCS broth at 220 rpm at 37 °C, when applicable the medium was supplemented with 100 µg/mL ampicillin. On the day of the infection, bacteria were sub-cultured from an overnight culture in fresh TCS at 1:100 dilution until OD_600_ reached ~0.45. Bacteria were harvested, washed once in EM buffer, and incubated for 15 min at 37 °C with shaking in EM buffer supplemented with 1 µg/mL poly-l-lysine hydrobromide (Sigma-Aldrich). The bacterial solution was washed twice, and the bacteria were diluted to the appropriate MOI.

### Congo Red induction assay

Congo Red effector protein secretion assay was carried out as described previously [58] with some modifications. Briefly, overnight bacterial cultures were diluted 1:50 and grown in fresh media as described previously. Bacterial suspensions were centrifuged, pellets resuspended in PBS to adjust 1 mL to an OD_600_ = 2, and incubated with or without 1% Congo Red (Sigma) at 37 °C with shaking (500 rpm) for 30 min. Samples were then centrifuged at 14,000*g* for 15 min at 4 °C. The supernatants were collected, and proteins precipitated for 30 min on ice using 1:1 (v/v) trichloroacetic acid at a final concentration of 20%. Proteins were recovered by centrifugation at 14,000*g* for 15 min at 4 °C and the pellets washed twice with ice-cold acetone. Protein samples were denatured in Laemmli buffer, boiled for 10 min at 95 °C and run in a NuPAGE 10% Bis–Tris Gel (Invitrogen) with a protein ladder (26619, Thermo Scientific). The gel was fixed with 50% methanol, 7% acetic acid for 15 min at RT, washed in milliQ water and stained with GelCode Blue Stain reagent (Themo Scientific) for 1 h. After destaining, gel was imaged using an iBright 1500 imaging system (Invitrogen).

### Fluorescence microscopy and immunolabeling

Imaging was carried out on a Nikon Ti-E inverted microscope equipped with a Perfect Focus System (PFS), a spinning disk confocal system (CSU-W1, Yokogawa), and an ORCA flash 4.0 camera (Hamamatsu). Time-lapse imaging was performed using a CFI S Plan Fluor ELWD 40×/0.60 air immersion objective (MRH08430, Nikon) and microscopy of fixed samples with a CFI Plan Apo VC 60×/1.2 water immersion objective (MRD07602, Nikon) or CFI Plan Apo 60×/1.4 oil immersion objective (MRD01605, Nikon). For fluorescent microscopy experiments, 40,000 cells were seeded into 8-well glass bottom microslides (ibidi). For time-lapse imaging, the microscope chamber was heated at 37 °C, and bacterial invasion was monitored immediately after bacteria addition to the cells. Images were recorded every minute for 2.5 h (*Shigella*) or 3.5 h *E. coli* mT3_*Shigella*_. For microscopy of fixed samples, cells were infected with *E. coli* strains at 37 °C, 5% CO_2_ for 2 h, while *Shigella* infections were carried out for 30 min at 37 °C. The inside-out staining protocol was adapted from another study [59]. Infected cells were washed three times in DPBS (Gibco) and fixed in 3.7% (wt/vol) Paraformaldehyde (PFA, Electron Microscopy Science), diluted in DPBS for 20 min at RT. All antibodies were diluted in an immunolabelling solution (2% BSA in DPBS) and incubated for 30 min at RT. First, the extracellular bacteria were labeled with a rabbit polyclonal antibody to *E. coli* (1:2,000; ab137967, Abcam) or a rabbit polyclonal antibody to *Shigella* (1:2,000; ab65282 Abcam). Cells were then washed three times with DPBS and permeabilized with 0.1% Triton X-100 in DPBS for 10 min at RT. Permeabilization solution was washed three times with DPBS. The extracellular bacteria were labeled with goat anti-rabbit AlexaFluor 647 (1:2,000; A32733, Invitrogen), and the total bacteria were stained with DAPI (1:2,000, Fisher Scientific). Finally, samples were washed three times with DPBS and stored hydrated at 4 °C, protected from light. z-stacks of individual positions were recorded with a 0.3 µm step size.

### Time-lapse image analysis

Images were analyzed using FIJI [60]. Images from the time-lapse series were corrected for the intensity decay due to photobleaching using the FIJI bleach correction plugin [61]. Vacuole damage and rupture times were manually quantified.

### *Shigella* epithelial cell infection for cryo-electron microscopy

Cryo-EM gold grids (Quantifoil R2/2 Au 200 mesh, Quantifoil) were placed in the slots of a custom-made PDMS stencil (Alvéole) on a 35 mm dish (µ-Dish 35 mm, ibidi). The montage was plasma-cleaned for 45 s and sterilized under UV irradiation in a laminar flow hood for 15 min. Cell culture medium was added to the dish and equilibrated for 15 min at 37 °C. 85,000–100,000 HeLa cells were seeded and grown for 24 h at 37 °C, 5% CO_2_. Before the infection, cells were washed three times with DMEM and incubated with bacterial suspension at a high MOI (100–400). Infection was synchronized at RT for 15 min and cells were infected at 37 °C for 10 or 20 min. Cells were washed three times with DPBS (Gibco) and fixed for 15 min in 2% PFA (Electron Microscopy Science), 0.05% Glutaraldehyde (Sigma-Aldrich) in 0.1 M HEPES (Gibco) followed by 15 min in 4% PFA, 0.1% Glutaraldehyde in 0.1 M HEPES. Fixed cells were washed three times with DPBS and immediately vitrified. Grids were blotted from the backside and plunged into liquid ethane at liquid nitrogen temperature using a Leica EM GP automatic plunger with the following settings: humidity 98%, blot time: 8 s, 20 °C. The vitrified grids were stored in sealed boxes in liquid nitrogen until further use.

### Fluorescence-guided cryo-FIB milling

Grids clipped into cryo-FIB milling Autogrids (Thermo Fisher Scientific) were imaged in light microscopy using a Leica THUNDER Imager EM Cryo-CLEM (Leica Microsystems). A global map of the grid was acquired to aid later correlations and individual z-stacks with 0.35 µm spacing were recorded at the regions of interest. Cryo-FIB lamellae were prepared using an Aquilos 2 dual-beam cryo-FIB scanning electron microscope (cryo-FIB-SEM; Thermo Fisher Scientific) instrument equipped with a cryo-transfer system, a cryo-stage, and a 45° pre-tilted shuttle (Thermo Fisher Scientific). In order to locate region of interest for the lamellae milling, an SEM map of the grid was acquired and cryo-fluorescence images were loaded at the correct relative position and orientation within the SEM map using Maps 3.2 (Thermo Fisher Scientific). Before milling an organometallic platinum layer was deposited on the cells for 1 min using the gas injection system. Lamellae were automatically milled overnight to 1 µm with a reducing current (1–0.1 nA) using AutoTEM (v.2.2, Thermo Fisher Scientific) with a 10° milling angle. The next day, lamellae were manually polished to ~200 nm and stored in liquid nitrogen.

### Cryo-ET sample preparation for bacterial cells

Bacteria were grown as described above and 1 mL of subculture was centrifuged at 6,010*g* for 2 min in a 2 mL Eppendorf tube. The bacterial pellet was washed twice in DPBS, then fixed, washed three times and resuspended in DPBS to an OD600 of 10. Immediately after resuspension, bacteria were plunge-frozen. To this end, 1 µ L of DPBS was applied on the backside of a glow-discharged copper grid (Quanifoil R2/2 Cu 200 mesh, Quanifoil), while 4 μL of the bacterial suspension was deposited on the film side and the grid was back-blotted for 4 seconds. Cryo-FIB milling was carried out as described above, without fluorescence-guided targeting.

### Tilt series data collection and reconstruction

Cryo-ET datasets were collected using SerialEM software [62] (version 4.1.0 to 4.1.4) on a 300 kV cold-Field Emission Gun Titan Krios transmission electron microscope (Thermo Fisher Scientific) equipped with a Falcon 4i direct electron detection camera (Thermo Fisher Scientific) and Selectris X an energy filter (Thermo Fisher Scientific). For tilt series acquisition the microscope was set in nanoprobe mode, energy filter at 20 eV (zero loss) and an objective aperture of 100. Tilt series were acquired with a dose dose-symmetric acquisition scheme [63] ranging from +70° to −50° starting from a 10° pretilt with 3° increments at the nominal magnification of 26,000× and 42,000× corresponding to a pixel size of 3.104 and 4.81 Å respectively with defocus ranging from 3 to 5 µm. The total dose applied was 140 e^−^/Å^2^ with an electron dose starting at 3.5 e^−^/Å^2^ at 10° pretilt and exposure time-varying to be 1.15 times higher at high tilts. The cold-FEG was flashed before each tilt series acquisition and frames were saved in eer format. Tilt series were processed with our in-house reconstruction script. First, the frames were motion-corrected and gain-corrected using MotionCor2 [64]. 2D contrast transfer function (CTF) was calculated with ctfplotter and the correction was applied with phase-flipping using ctfphaseflip [65]. Tilt series were dose-weighted and aligned in AreTomo [66] and tomograms were reconstructed using IMOD by weighted back-projection [67] to a pixel size ~10 Å corresponding to a binning of 2 or 3 depending on the dataset and applying and “exact filter”. Finally, reconstructed tomograms were filtered using isotropic reconstruction software IsoNet [68] with the following parameters (make_mask --density_percentage 50 --std_percentage 5; refine --iterations 30 --noise_start_iter 10,15,20,25 --noise_level 0.05,0.1,0.15,0.2). Tomograms presented in the supplementary movies were denoised with Topaz-Denoise [69].

### Tomogram analysis

#### Image correlation.

Post-acquisition image correlation was performed using a semi-automatic workflow. All images were placed into a world coordinate system by extracting their relative stage coordinates, pixel spacing and orientation from image metadata. First, precise spatial transformations between EM images at different resolutions were determined in an automated manner. For this, all EM montages were stitched using the python package multiview-stitcher [70]. Then, for each tomogram the map with the highest resolution containing its central coordinate was determined, i.e., a preview or anchor map. This map was then registered against its closest medium magnification map, which was subsequently registered against the low magnification map representing the grid level (using translation, rotation and uniform scaling). The so obtained transformations were then used to precisely place all EM images jointly onto the cryo-EM grid. Registration between the cryo-EM and fluorescent modalities was then performed at the grid level by estimating an affine transform from manually placed landmarks using the napari plugin affinder (https://github.com/jni/affinder). Finally, stacks containing transformed and cropped views of all relevant imaging modalities data around each tomogram position were generated at low, medium, and high resolutions and exported as composite tiff files. Python scripts related to this and other methods described in this publication are available under https://gitlab.pasteur.fr/iah/2024_swistak_et_al_code and make use of the scientific python ecosystem [71–77]. For figure preparation, channels of cryo-fLM images were registered using the FIJI Rigid Registration plugin [60] to correct for chromatic shift.

#### T3SS annotation and measurements.

IsoNet-filtered tomograms were manually annotated using IMOD 3D visualization tools (3dmod and slicer) [78]. Individual objects were created for each secretion system and points were placed in the inner membrane/basal body interface (point 1), basal body/needle junction or basal body/bacteria outer membrane junction for the basal body-only configuration (point 2), and the needle tip or vacuole membrane/T3SS tip interface (point 3) in this strict order. The obtained point coordinates were loaded into python using the package imodmodel (https://github.com/teamtomo/imodmodel). Distances between the T3SS points were calculated as the Euclidean distances between the manually determined 3D coordinates. For inter-T3SS distances, point 2 was considered as reference. Angles between the vectors $\vec{21\;}$ (from point 2 to point 1) and $\vec{23}$ were calculated to assess T3SSs base-to-needle geometry. Substacks around T3SSs were extracted by aligning their *y* axis with the vector $\vec{y^{\prime }\;}=\vec{23}$, the *x* axis with $\vec{x^{\prime }\;}=\vec{y^{\prime }}\;\times \;\vec{z}$ and the *z* axis with $\vec{x^{\prime }\;}=\;\vec{z^{\prime }\;}\times \vec{y^{\prime }}$, where $\vec{z\;}$ indicates the *z* axis of the tomogram and × the vector product.

#### Semi-automated segmentation.

Membranes of full tomograms or T3SSs substacks were automatically segmented using the MemBrain-Seg [79] segmentation tool (v2, v9b pretrained model). The resulting segmentations were imported into napari [80] for manual refinement. Segmented objects belonging to the inner and outer membranes of bacteria and vacuole membranes were annotated and manually curated to remove false positive detections. For display purposes, curated segmentations were loaded into UCSF ChimeraX [81] (v1.8), and *Shigella* T3SS atomic model (EMD-15700) was docked into our tomogram densities to indicate T3SS positions on bacterial surface.

#### Membrane morphometrics: Inter-membrane distance and curvedness calculation/representation.

For inter-membrane distance measurements, surface meshes were reconstructed from the connected components of the curated segmentation masks using the vtkSurfaceReconstruction filter of the Visualization Toolkit [82] within pyvista [83]. The distances between membranes were calculated by nearest-neighbor analysis. To avoid overestimating distances at surface edges, we excluded distance measurements for points with the nearest neighbor located at the edges of the reference surface. Instead, for those points, we considered the same distance as was assigned to the closest point within the same surface. Quantification of membrane curvedness[84] was performed using an open-source pipeline for surface morphometrics (https://github.com/GrotjahnLab/surface_morphometrics), which makes use of the curvature estimation package pycurv [34]. To avoid edge-induced surface reconstruction artifacts, we excluded regions of the membrane closer than 20 nm to the segmentation borders. 3D renderings for visualization were created using pyvista.

### Statistical analysis

Two-tailed unpaired *t*-tests and ANOVA with Tukey’s multiple comparisons tests were performed using GraphPad Prism version 10.2.2 for Windows and 10.4.1 for Mac, GraphPad Software, Boston, Massachusetts USA (www.graphpad.com). With a *p*-value considered significant if *p* < 0.05 with * < 0.05 and **** < 0.0001.

### Figure preparation

All figures were prepared using Adobe Illustrator (v. 27.8.1) and some elements (bacteria) of Figs 1A, 4, and S3 were created with BioRender.com.

## Supporting information

S1 Fig Ipa protein secretion profiles from the bacterial strains used in this study. SDS-PAGE showing secretion of Ipa proteins after Congo Red induction (+) or not (−). IpaA, B, C, and D migration bands are marked by asterisks. *Shigella* BS176 that does not carry the invasion plasmid encoding the T3SS does not secrete Ipa proteins. *E. coli* mT3_*Shigella*_ secretes IpaA, B, C and D. *E. coli* mT3_*Shigella*_Δeff_Inv that only encodes for T3SS, including needle tip protein (IpaD) and translocon pore proteins (IpaB and C) does not secrete the effector IpaA.
(TIF)

S2 Fig Synthetic *Escherichia coli* strains expressing *Shigella* T3SS invade epithelial cells with minimal effector set or *Yersinia* invasin expression. Representative microscopy images of HeLa eGFP-Lysenin mOrange-Galectin-3 cells infected with either DH10β *E. coli* or *E. coli* mT3_*Shigella*_, for 2 h, *E. coli* mT3_*Shigella*_Δeff_Inv for 3 h or WT *Shigella* for 30 min. Extracellular bacteria were stained with an antibody directed against LPS and total bacteria (extra and intracellular) were labeled with DAPI. The First panel shows full field of view, inset is marked by a dashed box. *E. coli* DH10β did not invade HeLa cells while *Shigella, E. coli* mT3_*Shigella*_ and *E. coli* mT3_*Shigella*_Δeff_Inv did. eGFP-Lysenin: yellow, mOrange-Galectin-3: magenta, DAPI: cyan, and α-LPS: green. Intracellular bacteria: cyan, Extracellular bacteria: cyan and green. Scale bars are 50 µm for the large field of view and 8 µm for all the insets.
(TIF)

S3 Fig Correlative cryo-ET recapitulates the successive steps of *Shigella* vacuole damage and rupture. **(A–C)** Detailed correlation steps for the identification intact (A), damaged (B), or ruptured (C) vacuoles (shown in Fig 2) in HeLa eGFP-Lysenin mOrange-Galectin-3 cells infected with Tag-BFP *Shigella* processed through a correlative cryo-ET workflow. (I) Vitrified cells on cryo-EM grids were imaged by cryo-fluorescence microscopy to localize infection sites and target them for lamella milling. Tag-BFP *Shigella*: cyan, eGFP-Lysenin: yellow, and mOrange-Galectin-3: magenta. Scale bars: 10 µm. (II) Cryo-TEM overview of the region targeted in I after cells were thinned into lamellae using cryo-FIB-milling. Scale bars 10 µm. (III) Cryo-lamellae maps overlayed with the corresponding cryo-fluorescence images. Scale bar 5 µm. The rotation angles from images I and II to III are indicated on the top left. (IV) Inset of III showing the bacteria that was targeted for imaging. Scale bar 1 µm. The rotation angles from images III to IV are indicated on the top left. Square boxes V correspond to regions presented in the tomographic slices of Fig 2A, 2B, and 2C respectively. **(D)** Additional T3SS insets marked in Fig 2 damaged and ruptured panels. Outlined arrowheads point to undetermined densities in the lumen of the *Shigella* vacuoles. Scale bars are 50 nm. Vac.: Vacuole, OM: Outer membrane, IM: Inner membrane.
(TIF)

S4 Fig Differential coating of *Shigella* vacuole upon loss of membrane integrity in cells constitutively expressing reporters of membrane damage and rupture. Slice through tomograms (scale bars 200 nm) and corresponding cryo-fLM images (scale bars 2 µm) of *Shigella* (white arrowhead) at different infection stages with vacuole membranes showing different coating patterns that may reflect on recruitment of overexpressed Lysenin (yellow arrowhead) and Galectin-3 (magenta arrowhead). Intact vacuoles: membrane coating is never observed. Damaged vacuoles: cytosolic side of the vacuole membrane uniformly coated with electron-dense layers upon Lysenin recruitment. Ruptured vacuoles: both on the cytosolic and luminal side of Lysenin and Galectin-3 double-positive vacuoles are coated while only the luminal side of the vacuole membrane is coated in cells expressing just the Galectin-3 marker.
(TIF)

S5 Fig T3SS complexes are reliably found in T3SS-expressing bacterial strains. Cryo-tomograms were acquired from cryo-lamellae of various bacterial strains, with T3SS-like complexes identified exclusively in the tomograms of T3SS-encoding strains. Tomographic slices are shown (scale bars 200 nm) and when applicable T3SS regions are marked by a dashed box and corresponding insets shown (scale bars 50 nm). Z slices of tomograms and insets are indicated below the images. Vac.: Vacuole, OM: Outer membrane, IM: Inner membrane. **(A)** *Shigella flexneri* BS1276 (pWR100 deficient strain). No T3SSs were seen in tomograms. **(B)** WT *Shigella flexneri* M90T carrying the virulence plasmid pWR100, encoding for the T3SS structural component and most of the *Shigella* secreted effectors. Note that inset n°1 shows a gray area as the T3SS is on the edge of the tomogram. **(C)** *E. coli* mT3_*Shigella*_, engineered *E. coli* strain expressing *Shigella* T3SS structural components including the translocon pore (IpaB, IpaC) and secreted effectors (IpaA, IcsB, IpgD).
(TIF)

S6 Fig T3SS assemblies along *Shigella* infection stages. **(A)** Diversity of T3SS architectures might reflect different assembly states. Contingency graph of the percentage of T3SSs with a membrane-spanning basal body and protruding needle (total *n* = 122) or with only a basal body without needles (total *n* = 25) plotted according to the infection stage. Representative examples of putative T3SSs assembly states are shown. Scale bars are 50 nm. The data underlying S6 Fig can be found in S4 Data. **(B)** T3SS needles are exposed to the cytosol after vacuole rupture and disassembly. Cryo-fLM, (scale bars 2 µm) and corresponding tomogram slice (scale bar 200 nm) of *Shigella* with ruptured vacuole displaying T3SSs with needles exposed to the cytosol after vacuole rupture (insets, scale bars 50 nm). OM: Outer membrane, IM: Inner membrane.
(TIF)

S7 Fig T3SS-vacuole contact depends on both T3SS length and available luminal space. **(A–D)** Tomogram slices of the T3SS-vacuole zones before *Shigella* membrane rupture. White arrowhead points to bacterial membranes deformations. Scale bars 50 nm. Corresponding 3D rendering of vacuole and bacteria membranes with *Shigella* T3SS 3D map (EMD-15700) fitted. Galectin-3 negative vacuole (gray), bacteria OM (dark blue) and IM (light blue). Next panels show membrane surface reconstructions of the vacuole membrane coloured by 3D distance to the bacteria outer membrane (left) or vacuole membrane curvedness (right). Bacteria membranes are shown in light gray. Vac.: Vacuole, OM: Outer membrane, IM: Inner membrane. The data underlying S7 Fig can be found at https://doi.org/10.5281/zenodo.15065516. (A and B) T3SSs do not establish contact with the vacuole if they are short (A) or if the vacuole is locally relaxed (B). (C) T3SS with needle length correlating with the available vacuole space contact the vacuole without deforming it. (D) T3SS with long needle contacting the vacuole membrane adopts tilted insertion within bacterial membranes. **(E)** Quantification of the vacuole membrane curvedness (nm^−1^) as a function of the distance from the T3SS needle tip (exemplified in F), depending on whether or not T3SS establishes contact with the vacuole (T3SS tips with a distance smaller than 5 nm were considered to be in contact). Bars show the 5%–95% percentile range. **(F)** Annotated graphical representation showing curvedness values and their distances to the T3SS needle tip in bins of width 10 nm, as used for the quantification in (E).
(TIF)

S8 Fig T3SS-vacuole contact depends on both T3SS length and available luminal space. Correlations of the tomogram insets shown in Figs 4 and S7. See also S7–S9 Videos. Slice through tomograms (scale bars 200 nm) and corresponding cryo-fLM images (scale bars 2 µm) of *Shigella* (white arrowhead) before vacuole rupture. Inset correspondence: **(A)** T3SS 1: S7B Fig, 2: Fig 4B, 3: S7D Fig, 4: Fig 4A. **(B)** T3SS 1: Fig 4C, 2: S7D Fig, 3: S7A Fig. **(C)** T3SSs 1, 2 and 3: Fig 4D.
(TIF)

S1 Video Time-lapse microscopy monitoring of Lysenin and Galectin-3 recruitment at the *Shigella* vacuole, related to Fig 1B. *Shigella* infection of HeLa eGFP-Lysenin mOrange-Galectin-3 cells. Vacuolar damage characterized by Lysenin recruitment at the vacuole (merge: yellow, grayscale: second panel) is observed shortly before vacuolar rupture (merge: magenta, grayscale: third panel) indicated by the recruitment of Galectin-3 at the vacuole and is followed by vacuole disassembly. Images were taken every minute and a maximum intensity z-projection is presented. Scale bar 5 µm.
(MP4)

S2 Video Time-lapse microscopy monitoring of Lysenin and Galectin-3 recruitment at the *Escherichia coli* mT3_*Shigella*_ vacuole, related to Fig 1D. *E. coli* mT3_*Shigella*_ infection of HeLa eGFP-Lysenin mOrange-Galectin-3 cells. Vacuolar damage characterized by Lysenin recruitment at the vacuole (merge: yellow, grayscale: second panel) is observed before vacuolar rupture (merge: magenta, grayscale: third panel) indicated by the recruitment of Galectin-3 at the vacuole that does not disassemble. Images were taken every minute and a maximum intensity z-projection is presented. Scale bar 5 µm.
(MP4)

S3 Video Time-lapse microscopy monitoring of Lysenin and Galectin-3 recruitment at the *Escherichia coli E. coli* mT3_*Shigella*_Δeff_Inv vacuole, related to Fig 1F. *E. coli* mT3_*Shigella*_Δeff_Inv infection of HeLa eGFP-Lysenin mOrange-Galectin-3 cells. Vacuolar damage characterized by Lysenin recruitment at the vacuole (merge: yellow, grayscale: second panel) is observed very shortly before vacuolar rupture (merge: magenta, grayscale: third panel) indicated by the recruitment of Galectin-3 at the vacuole that does not disassemble. Images were taken every minute and a maximum intensity z-projection is presented. Scale bar 5 µm.
(MP4)

S4 Video Tomogram and corresponding segmentations of the distinct stages of *Shigella* cytosolic access, related to Fig 2A. Sequential slices through the tomogram shown in Fig 2A and segmentation showing *Shigella* entrapped into an intact vacuole. *Shigella* OM: Outer membrane (dark blue), IM: Inner membrane (light blue). Intact vacuole (gray. Scale bar 200 nm.
(MOV)

S5 Video Tomogram and corresponding segmentations of *Shigella* in a damaged vacuole, related to Fig 2B. Sequential slices through the tomogram shown in Fig 2B and segmentation showing *Shigella* entrapped either into a damaged vacuole. *Shigella* OM: Outer membrane (dark blue), IM: Inner membrane (light blue). Damaged vacuole (yellow). Scale bar 200 nm.
(MOV)

S6 Video Tomogram and corresponding segmentations of *Shigella* cytosolic access, related to Fig 2C. Sequential slices through the tomogram shown in Fig 2C and segmentation showing *Shigella* entrapped either into an intact or damaged vacuole or surrounded by ruptured vacuole remnants. *Shigella* OM: Outer membrane (dark blue), IM: Inner membrane (light blue). Ruptured vacuole (magenta). Scale bar 200 nm.
(MOV)

S7 Video Tomogram and corresponding segmentations of *Shigella* prior cytosolic access, related to S8A Fig. Sequential slices through the tomogram of S8A Fig with corresponding segmentations showing *Shigella* in a Galectin-3 negative vacuole. *Shigella* OM: Outer membrane (dark blue), IM: Inner membrane (light blue), Galectin-3 negative vacuole (gray). Scale bar 200 nm.
(MOV)

S8 Video Tomogram and corresponding segmentations of *Shigella* prior cytosolic access, related to S8B Fig. Sequential slices through the tomogram of S8B Fig with corresponding segmentations showing *Shigella* in a Galectin-3 negative vacuoles. *Shigella* OM: Outer membrane (dark blue), IM: Inner membrane (light blue), Galectin-3 negative vacuole (gray). Scale bar 200 nm.
(MOV)

S9 Video Tomogram and corresponding segmentation of *Shigella* prior cytosolic access, related to S8C Fig. Sequential slices through the tomogram of S8C Fig with corresponding segmentations showing *Shigella* in a damaged vacuole. *Shigella* OM: Outer membrane (dark blue), IM: Inner membrane (light blue), damaged vacuole (yellow). Scale bars are 200 nm.
(MOV)

S10 Video T3SS punctures the host endomembrane, related to Fig 4C. Sequential slices through a zone of tomogram showing a T3SS puncturing the vacuolar membrane. Scale bar is 50 nm.
(MOV)

S1 Data Data underlying Fig 1. (XLSX)

S2 Data Data underlying Fig 2. (XLSX)

S3 Data Data underlying Fig 3. (XLSX)

S4 Data Data underlying S6 Fig. (XLSX)

S1 Raw Image Data underlying S1 Fig. (PDF)

## Abbreviations

cryo-CLEM: cryo-correlative light and electron microscopy
cryo-ET: cryo-electron tomography
cryo-FIB: cryo-focused ion beam
cryo-fLM: cryo-fluorescence microscopy
CTF: contrast transfer function
DMEM: Dulbecco’s modified Eagle’s medium
MOI: multiplicity of infection
RT: room temperature
TCS: trypticase soy
T3SS: Type Three Secretion System
3D: three dimensions

## References

[1] Creasey EA, Isberg RR. Maintenance of vacuole integrity by bacterial pathogens. Curr Opin Microbiol. 2014;17:46–52. doi: 10.1016/j.mib.2013.11.005 24581692 PMC4009691

[2] Weddle E, Agaisse H. Principles of intracellular bacterial pathogen spread from cell to cell. PLOS Pathog. 2018;14:e1007380. doi: 10.1371/journal.ppat.1007380 30543716 PMC6292572

[3] Gutierrez MG, Enninga J. Intracellular niche switching as host subversion strategy of bacterial pathogens. Curr Opin Cell Biol. 2022;76:102081. doi: 10.1016/j.ceb.2022.102081 35487154

[4] Petrišič N, Kozorog M, Aden S, Podobnik M, Anderluh G. The molecular mechanisms of listeriolysin O-induced lipid membrane damage. Biochim Biophys Acta Biomembr. 2021;1863:183604. doi: 10.1016/j.bbamem.2021.183604 33722646

[5] Augenstreich J, Haanappel E, Ferré G, Czaplicki G, Jolibois F, Destainville N, et al. The conical shape of DIM lipids promotes Mycobacterium tuberculosis infection of macrophages. Proc Natl Acad Sci U S A. 2019;116:25649–58. doi: 10.1073/pnas.1910368116 31757855 PMC6926010

[6] Barker JR, Chong A, Wehrly TD, Yu J-J, Rodriguez SA, Liu J, et al. The Francisella tularensis pathogenicity island encodes a secretion system that is required for phagosome escape and virulence. Mol Microbiol. 2009;74:1459–70. doi: 10.1111/j.1365-2958.2009.06947.x 20054881 PMC2814410

[7] Hsu T, Hingley-Wilson SM, Chen B, Chen M, Dai AZ, Morin PM, et al. The primary mechanism of attenuation of bacillus Calmette-Guerin is a loss of secreted lytic function required for invasion of lung interstitial tissue. Proc Natl Acad Sci U S A. 2003;100:12420–5. doi: 10.1073/pnas.1635213100 14557547 PMC218773

[8] Simeone R, Bobard A, Lippmann J, Bitter W, Majlessi L, Brosch R, et al. Phagosomal rupture by Mycobacterium tuberculosis results in toxicity and host cell death. PLOS Pathogens. 2012;8:e1002507. doi: 10.1371/journal.ppat.1002507 22319448 PMC3271072

[9] Barisch C, Holthuis JCM, Cosentino K. Membrane damage and repair: a thin line between life and death. Biol Chem. 2023;404:467–90. doi: 10.1515/hsz-2022-0321 36810295

[10] Schnupf P, Sansonetti PJ. Shigella pathogenesis: new insights through advanced methodologies. Microbiol Spectr. 2019:15–39. doi: 10.1128/microbiolspec.bai-0023-2019 30953429 PMC11588159

[11] Weiner A, Mellouk N, Lopez-Montero N, Chang YY, Souque C, Schmitt C, et al. Macropinosomes are key players in early Shigella invasion and vacuolar escape in epithelial cells. PLoS Pathog. 2016;12:e1005602. doi: 10.1371/journal.ppat.1005602 27182929 PMC4868309

[12] Chang Y-Y, Valenzuela C, Lensen A, Lopez-Montero N, Sidik S, Salogiannis J, et al. Microtubules provide force to promote membrane uncoating in vacuolar escape for a cyto-invasive bacterial pathogen. Nat Commun. 2024;15:1065. doi: 10.1038/s41467-024-45182-6 38316786 PMC10844605

[13] Kühn S, Bergqvist J, Gil M, Valenzuela C, Barrio L, Lebreton S, et al. Actin assembly around the Shigella-containing vacuole promotes successful infection. Cell Rep. 2020;31:107638. doi: 10.1016/J.CELREP.2020.107638 32402280 PMC7225751

[14] Du J, Reeves AZ, Klein JA, Twedt DJ, Knodler LA, Lesser CF. The type III secretion system apparatus determines the intracellular niche of bacterial pathogens. Proc Natl Acad Sci U S A. 2016;113:4794–9. doi: 10.1073/pnas.1520699113 27078095 PMC4855615

[15] Deng W, Marshall NC, Rowland JL, McCoy JM, Worrall LJ, Santos AS, et al. Assembly, structure, function and regulation of type III secretion systems. Nat Rev Microbiol. 2017;15:323–37. doi: 10.1038/nrmicro.2017.20 28392566

[16] Blocker A, Gounon P, Larquet E, Niebuhr K, Cabiaux V, Parsot C, et al. The tripartite type III secreton of Shigella flexneri inserts IpaB and IpaC into host membranes. J Cell Biol. 1999;147(3):683–93. doi: 10.1083/jcb.147.3.683 10545510 PMC2151192

[17] Ellison CJ, Kukulski W, Boyle KB, Munro S, Randow F. Transbilayer movement of sphingomyelin precedes catastrophic breakage of enterobacteria-containing vacuoles. Curr Biol. 2020;30:2974–2983.e6. doi: 10.1016/j.cub.2020.05.083 32649908 PMC7416114

[18] Paz I, Sachse M, Dupont N, Mounier J, Cederfur C, Enninga J, et al. Galectin-3, a marker for vacuole lysis by invasive pathogens. Cellular Microbiol. 2010;12:530–44. doi: 10.1111/j.1462-5822.2009.01415.x 19951367

[19] Reeves AZ, Spears WE, Du J, Tan KY, Wagers AJ, Lesser CF. Engineering Escherichia coli into a protein delivery system for mammalian cells. ACS Synth Biol. 2015;4:644–54. doi: 10.1021/acssynbio.5b00002 25853840 PMC4487226

[20] Isberg RR, Falkow S. A single genetic locus encoded by Yersinia pseudotuberculosis permits invasion of cultured animal cells by Escherichia coli K-12. Nature. 1985;317:262–4. doi: 10.1038/317262a0 2995819

[21] Mori T, Niki T, Uchida Y, Mukai K, Kuchitsu Y, Kishimoto T, et al. A non-toxic equinatoxin-II reveals the dynamics and distribution of sphingomyelin in the cytosolic leaflet of the plasma membrane. Sci Rep. 2024;14:16872. doi: 10.1038/s41598-024-67803-2 39043900 PMC11266560

[22] Jacobson K, Liu P, Lagerholm BC. The lateral organization and mobility of plasma membrane components. Cell. 2019;177:806–19. doi: 10.1016/j.cell.2019.04.018 31051105 PMC6541401

[23] Keck C, Enninga J, Swistak L. Caught in the act: in situ visualization of bacterial secretion systems by cryo-electron tomography. Mol Microbiol. 2023;121:636–45. doi: 10.1111/mmi.15186 37975530

[24] Wagner FR, Watanabe R, Schampers R, Singh D, Persoon H, Schaffer M, et al. Preparing samples from whole cells using focused-ion-beam milling for cryo-electron tomography. Nat Protoc. 2020;15:2041–70. doi: 10.1038/s41596-020-0320-x 32405053 PMC8053421

[25] Shetty A, Chen S, Tocheva EI, Jensen GJ, Hickey WJ. Nanopods: a new bacterial structure and mechanism for deployment of outer membrane vesicles. PLOS ONE. 2011;6:e20725. doi: 10.1371/journal.pone.0020725 21687732 PMC3110197

[26] Kaplan M, Chreifi G, Metskas LA, Liedtke J, Wood CR, Oikonomou CM, et al. In situ imaging of bacterial outer membrane projections and associated protein complexes using electron cryo-tomography. eLife. 2021;10:e73099. doi: 10.7554/eLife.73099 34468314 PMC8455137

[27] Chang Y-Y, Stévenin V, Duchateau M, Giai Gianetto Q, Hourdel V, Rodrigues CD, et al. Shigella hijacks the exocyst to cluster macropinosomes for efficient vacuolar escape. PLOS Pathogens. 2020;16:e1008822. doi: 10.1371/journal.ppat.1008822 32866204 PMC7485983

[28] Jaumouillé V, Francetic O, Sansonetti PJ, Tran Van Nhieu G. Cytoplasmic targeting of IpaC to the bacterial pole directs polar type III secretion in Shigella. EMBO J. 2008;27:447–57. doi: 10.1038/sj.emboj.7601976 18188151 PMC2234337

[29] Nans A, Saibil HR, Hayward RD. Pathogen-host reorganization during Chlamydia invasion revealed by cryo-electron tomography. Cell Microbiol. 2014;16:1457–72. doi: 10.1111/cmi.12310 24809274 PMC4336559

[30] Guo EZ, Galán JE. Cryo-EM structure of the needle filament tip complex of the Salmonella type III secretion injectisome. Proc Natl Acad Sci. 2021;118:e2114552118. doi: 10.1073/pnas.2114552118 34706941 PMC8612237

[31] Naseer N, Zhang J, Bauer R, Constant DA, Nice TJ, Brodsky IE, et al. Salmonella enterica Serovar Typhimurium Induces NAIP/NLRC4- and NLRP3/ASC-Independent, Caspase-4-Dependent Inflammasome Activation in Human Intestinal Epithelial Cells. Infect Immun. 2022;90:e00663-21. doi: 10.1128/iai.00663-21 35678562 PMC9302179

[32] Campbell-Valois F-X, Schnupf P, Nigro G, Sachse M, Sansonetti PJ, Parsot C. A fluorescent reporter reveals on/off regulation of the Shigella type III secretion apparatus during entry and cell-to-cell spread. Cell Host Microbe. 2014;15:177–89. doi: 10.1016/j.chom.2014.01.005 24528864

[33] Tamano K, Katayama E, Toyotome T, Sasakawa C. Shigella Spa32 is an essential secretory protein for functional type III secretion machinery and uniformity of its needle length. J Bacteriol. 2002;184(5):1244–52. doi: 10.1128/JB.184.5.1244-1252.2002 11844752 PMC134865

[34] Salfer M, Collado JF, Baumeister W, Fernández-Busnadiego R, Martínez-Sánchez A. Reliable estimation of membrane curvature for cryo-electron tomography. PLOS Comput Biol. 2020;16:e1007962. doi: 10.1371/journal.pcbi.1007962 32776920 PMC7444595

[35] Barbee KA. Mechanical cell injury. Ann N Y Acad Sci. 2006;1066:67–84. doi: 10.1196/annals.1363.006 16533919

[36] Rudolph M, Carsten A, Kulnik S, Aepfelbacher M, Wolters M. Live imaging of Yersinia translocon formation and immune recognition in host cells. PLOS Pathogens. 2022;18:e1010251. doi: 10.1371/journal.ppat.1010251 35604950 PMC9173619

[37] Park D, Lara-Tejero M, Waxham MN, Li W, Hu B, Galán JE, et al. Visualization of the type III secretion mediated Salmonella-host cell interface using cryo-electron tomography. eLife. 2018;7:e39514. doi: 10.7554/eLife.39514 30281019 PMC6175578

[38] Tran N, Serfis AB, Osiecki JC, Picking WL, Coye L, Davis R, et al. Interaction of Shigella flexneri IpaC with model membranes correlates with effects on cultured cells. Infect Immun. 2000;68:3710–15. doi: 10.1128/IAI.68.6.3710-3715.2000 10816532 PMC97663

[39] Dickenson NE, Choudhari SP, Adam PR, Kramer RM, Joshi SB, Middaugh CR, et al. Oligomeric states of the Shigella translocator protein IpaB provide structural insights into formation of the type III secretion translocon. Protein Sci. 2013;22:614–27. doi: 10.1002/pro.2245 23456854 PMC3649263

[40] Journet L, Agrain C, Broz P, Cornelis GR. The needle length of bacterial injectisomes is determined by a molecular ruler. Science. 2003;302:1757–60. doi: 10.1126/science.1091422 14657497

[41] Mota LJ, Journet L, Sorg I, Agrain C, Cornelis GR. Bacterial injectisomes: Needle length does matter. Science. 2005;307:1278. doi: 10.1126/science.1107679 15731447

[42] Roduit C, van der Goot FG, De Los Rios P, Yersin A, Steiner P, Dietler G, et al. Elastic membrane heterogeneity of living cells revealed by stiff nanoscale membrane domains. Biophys J. 2008;94:1521–32. doi: 10.1529/biophysj.107.112862 17981897 PMC2212673

[43] Lafont F, Tran Van Nhieu G, Hanada K, Sansonetti P, van der Goot FG. Initial steps of Shigella infection depend on the cholesterol/sphingolipid raft-mediated CD44-IpaB interaction. EMBO J. 2002;21:4449–57. doi: 10.1093/emboj/cdf457 12198147 PMC126195

[44] Berger C, Ravelli RBG, López-Iglesias C, Kudryashev M, Diepold A, Peters PJ. Structure of the Yersinia injectisome in intracellular host cell phagosomes revealed by cryo FIB electron tomography. J Struct Biol. 2021;213:107701. doi: 10.1016/j.jsb.2021.107701 33549695

[45] van Ooij C, Apodaca G, Engel J. Characterization of the Chlamydia trachomatis vacuole and its interaction with the host endocytic pathway in HeLa cells. Infect Immun. 1997;65:758–66. doi: 10.1128/iai.65.2.758-766.1997 9009339 PMC176122

[46] Horwitz MA. Formation of a novel phagosome by the Legionnaires’ disease bacterium (Legionella pneumophila) in human monocytes. J Exp Med. 1983;158:1319–31. doi: 10.1084/jem.158.4.1319 6619736 PMC2187375

[47] Kumar Y, Valdivia RH. Actin and intermediate filaments stabilize the Chlamydia trachomatis vacuole by forming dynamic structural scaffolds. Cell Host Microbe. 2008;4:159–69. doi: 10.1016/j.chom.2008.05.018 18692775 PMC2605408

[48] Bujny MV, Ewels PA, Humphrey S, Attar N, Jepson MA, Cullen PJ. Sorting nexin-1 defines an early phase of Salmonella-containing vacuole-remodeling during Salmonella infection. J Cell Sci. 2008;121:2027–36. doi: 10.1242/jcs.018432 18505799

[49] Braun V, Wong A, Landekic M, Hong WJ, Grinstein S, Brumell JH. Sorting nexin 3 (SNX3) is a component of a tubular endosomal network induced by Salmonella and involved in maturation of the Salmonella-containing vacuole. Cell Microbiol. 2010;12:1352–67. doi: 10.1111/j.1462-5822.2010.01476.x 20482551

[50] Stévenin V, Chang Y-Y, Le Toquin Y, Duchateau M, Gianetto QG, Luk CH, et al. Dynamic growth and shrinkage of the salmonella-containing vacuole determines the intracellular pathogen niche. Cell Rep. 2019;29:3958–3973.e7. doi: 10.1016/j.celrep.2019.11.049 31851926 PMC6931108

[51] Kreibich S, Emmenlauer M, Fredlund J, Rämö P, Münz C, Dehio C, et al. Autophagy proteins promote repair of endosomal membranes damaged by the salmonella type three secretion system 1. Cell Host Microbe. 2015;18(5):527–37. doi: 10.1016/j.chom.2015.10.015 26567507

[52] Luk CH, Yu W, Deriano L, Enninga J. Salmonella subverts autophagy balancing bacterial fate and cellular inflammation. bioRxiv. 2020. p. 2020.11.15.383372. doi: 10.1101/2020.11.15.383372

[53] Cooper ST, McNeil PL. Membrane repair: mechanisms and pathophysiology. Physiol Rev. 2015;95:1205–40. doi: 10.1152/physrev.00037.2014 26336031 PMC4600952

[54] Plum MTW, Cheung HC, Iscar PR, Chen Y, Gan Y-H, Basler M. Burkholderia thailandensis uses a type VI secretion system to lyse protrusions without triggering host cell responses. Cell Host Microbe. 2024;32:676-692.e5. doi: 10.1016/j.chom.2024.03.013 38640929

[55] Kowarz E, Löscher D, Marschalek R. Optimized sleeping beauty transposons rapidly generate stable transgenic cell lines. Biotechnol J. 2015;10:647–53. doi: 10.1002/biot.201400821 25650551

[56] Mellouk N, Lensen A, Lopez-Montero N, Gil M, Valenzuela C, Klinkert K, et al. Post-translational targeting of Rab35 by the effector IcsB of Shigella determines intracellular bacterial niche formation. Cell Rep. 2024;43:114034. doi: 10.1016/j.celrep.2024.114034 38568808

[57] Sansonetti PJ, Mounier J. Metabolic events mediating early killing of host cells infected by Shigella flexneri. Microb Pathog. 1987;3:53–61. doi: 10.1016/0882-4010(87)90037-4 2848171

[58] Bahrani FK, Sansonetti PJ, Parsot C. Secretion of Ipa proteins by Shigella flexneri: inducer molecules and kinetics of activation. Infect Immun. 1997;65:4005–10. doi: 10.1128/iai.65.10.4005-4010.1997 9316999 PMC175575

[59] Duménil G, Olivo JC, Pellegrini S, Fellous M, Sansonetti PJ, Nhieu GT. Interferon α inhibits a Src-mediated pathway necessary for Shigella-induced cytoskeletal rearrangements in epithelial cells. J Cell Biol. 1998;143(4):1003–12. doi: 10.1083/jcb.143.4.1003 9817757 PMC2132965

[60] Schindelin J, Arganda-Carreras I, Frise E, Kaynig V, Longair M, Pietzsch T, et al. Fiji: an open-source platform for biological-image analysis. Nat Methods. 2012;9:676–82. doi: 10.1038/nmeth.2019 22743772 PMC3855844

[61] Miura K. Bleach correction ImageJ plugin for compensating the photobleaching of time-lapse sequences. F1000Res. 2020;9:1494. doi: 10.12688/f1000research.27171.1 33633845 PMC7871415

[62] Mastronarde DN. Automated electron microscope tomography using robust prediction of specimen movements. J Struct Biol. 2005;152:36–51. doi: 10.1016/j.jsb.2005.07.007 16182563

[63] Hagen WJH, Wan W, Briggs JAG. Implementation of a cryo-electron tomography tilt-scheme optimized for high resolution subtomogram averaging. J Struct Biol. 2017;197:191–8. doi: 10.1016/j.jsb.2016.06.007 27313000 PMC5287356

[64] Zheng SQ, Palovcak E, Armache J-P, Verba KA, Cheng Y, Agard DA. MotionCor2: anisotropic correction of beam-induced motion for improved cryo-electron microscopy. Nat Methods. 2017;14:331–2. doi: 10.1038/nmeth.4193 28250466 PMC5494038

[65] Xiong Q, Morphew MK, Schwartz CL, Hoenger AH, Mastronarde DN. CTF determination and correction for low dose tomographic tilt series. J Struct Biol. 2009;168:378–87. doi: 10.1016/j.jsb.2009.08.016 19732834 PMC2784817

[66] Zheng S, Wolff G, Greenan G, Chen Z, Faas FGA, Bárcena M, et al. AreTomo: an integrated software package for automated marker-free, motion-corrected cryo-electron tomographic alignment and reconstruction. J Struct Biol. 2022;6:100068. doi: 10.1016/j.yjsbx.2022.100068 35601683 PMC9117686

[67] Mastronarde DN, Held SR. Automated tilt series alignment and tomographic reconstruction in IMOD. J Struct Biol. 2017;197:102–13. doi: 10.1016/j.jsb.2016.07.011 27444392 PMC5247408

[68] Liu Y-T, Zhang H, Wang H, Tao C-L, Bi G-Q, Zhou ZH. Isotropic reconstruction for electron tomography with deep learning. Nat Commun. 2022;13:6482. doi: 10.1038/s41467-022-33957-8 36309499 PMC9617606

[69] Bepler T, Kelley K, Noble AJ, Berger B. Topaz-Denoise: general deep denoising models for cryoEM and cryoET. Nat Commun. 2020;11:5208. 10.1038/s41467-020-18952-1 33060581 PMC7567117

[70] Albert M, Michaut A, Wilhelmi A, Golden A, Tinevez J-Y. An extensible python toolbox for scalable image registration and fusion. Zenodo;2024. doi: 10.5281/zenodo.13151253

[71] Harris CR, Millman KJ, van der Walt SJ, Gommers R, Virtanen P, Cournapeau D, et al. Array programming with NumPy. Nature. 2020;585(7825):357–62. doi: 10.1038/s41586-020-2649-2 32939066 PMC7759461

[72] Virtanen P, Gommers R, Oliphant TE, Haberland M, Reddy T, Cournapeau D, et al. SciPy 1.0: fundamental algorithms for scientific computing in Python. Nat Methods. 2020;17:261–72. doi: 10.1038/s41592-019-0686-2 32015543 PMC7056644

[73] McKinney W. Data structures for statistical computing in Python. Proceedings of the 9th Python in Science Conference. 2010. p. 56–61. doi: 10.25080/Majora-92bf1922-00a

[74] van der Walt S, Schönberger JL, Nunez-Iglesias J, Boulogne F, Warner JD, Yager N, et al. scikit-image: image processing in Python. PeerJ. 2014;2:e453. doi: 10.7717/peerj.453 25024921 PMC4081273

[75] Burnley T, Palmer CM, Winn M. Recent developments in the CCP-EM software suite. Acta Cryst D. 2017;73:469–77. doi: 10.1107/S2059798317007859 28580908 PMC5458488

[76] Perez F, Granger BE. IPython: a system for interactive scientific computing. Comput Sci Eng. 2007;9:21–9. doi: 10.1109/MCSE.2007.53

[77] Brown EM, Toloudis D, Sherman J, Swain-Bowden M, Lambert T, AICSImageIO Contributors. AICSImageIO: image reading, metadata conversion, and image writing for microscopy images in pure python. GitHub; 2021. Available from: https://github.com/AllenCellModeling/aicsimageio

[78] Kremer JR, Mastronarde DN, McIntosh JR. Computer visualization of three-dimensional image data using IMOD. J Struct Biol. 1996;116:71–6. doi: 10.1006/jsbi.1996.0013 8742726

[79] Lamm L, Zufferey S, Righetto RD, Wietrzynski W, Yamauchi KA, Burt A, et al. MemBrain v2: an end-to-end tool for the analysis of membranes in cryo-electron tomography. bioRxiv. 2024. p. 2024.01.05.574336. doi: 10.1101/2024.01.05.574336 42711494

[80] Sofroniew N, Lambert T, Bokota G, Nunez-Iglesias J, Sobolewski P, Sweet A, et al. napari: a multi-dimensional image viewer for Python. Zenodo; 2024. doi: 10.5281/zenodo.12805667

[81] Pettersen EF, Goddard TD, Huang CC, Meng EC, Couch GS, Croll TI, et al. UCSF ChimeraX: Structure visualization for researchers, educators, and developers. Protein Sci. 2021;30:70–82. doi: 10.1002/pro.3943 32881101 PMC7737788

[82] Schroeder W, Martin K, Lorensen B. The Visualization Toolkit (4th ed.). Kitware; 2006.

[83] Sullivan C, Kaszynski A. PyVista: 3D plotting and mesh analysis through a streamlined interface for the Visualization Toolkit (VTK). J Open Source Softw. 2019;4:1450. 10.21105/joss.01450

[84] Koenderink JJ, van Doorn AJ. Surface shape and curvature scales. Image Vision Comput. 1992;10:557–64. doi: 10.1016/0262-8856(92)90076-F

