# Supplementary figures and images for: The bacterial type three secretion system induces mechanoporation of vacuolar membranes
Source: PLoS Biol. 2025 May 1;23(5):e3003135. doi: 10.1371/journal.pbio.3003135 (PMC12045489; doi:10.1371/journal.pbio.3003135)

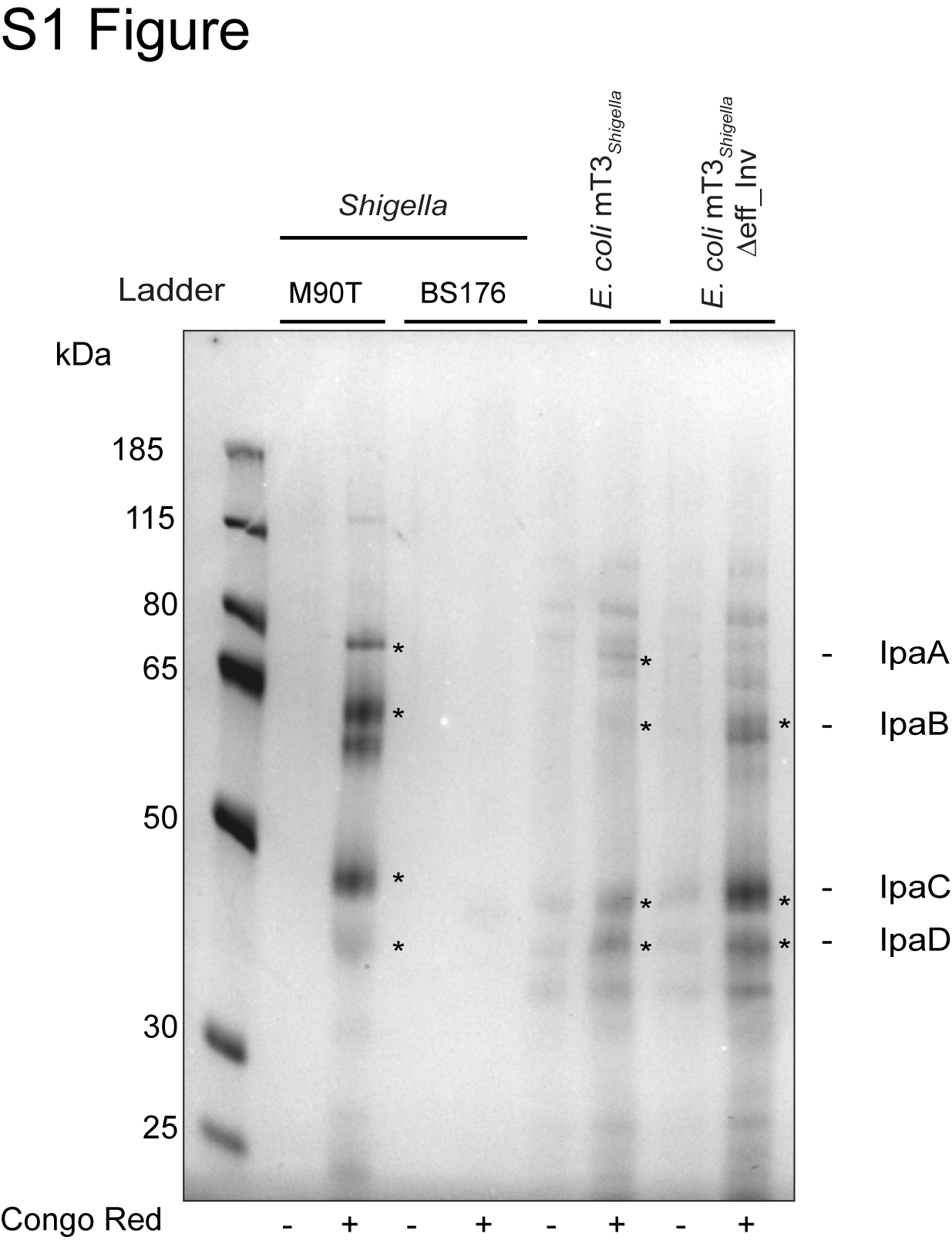

Supplement: S1 Fig — SDS-PAGE showing secretion of Ipa proteins after Congo Red induction (+) or not (−). IpaA, B, C, and D migration bands are marked by asterisks. Shigella BS176 that does not carry the invasion plasmid encoding the T3SS does not secrete Ipa proteins. E. coli mT3Shigella secretes IpaA, B, C and D. E. coli mT3ShigellaΔeff_Inv that only encodes for T3SS, including needle tip protein (IpaD) and translocon pore proteins (IpaB and C) does not secrete the effector IpaA. (TIF) [file pbio.3003135.s001.tif]

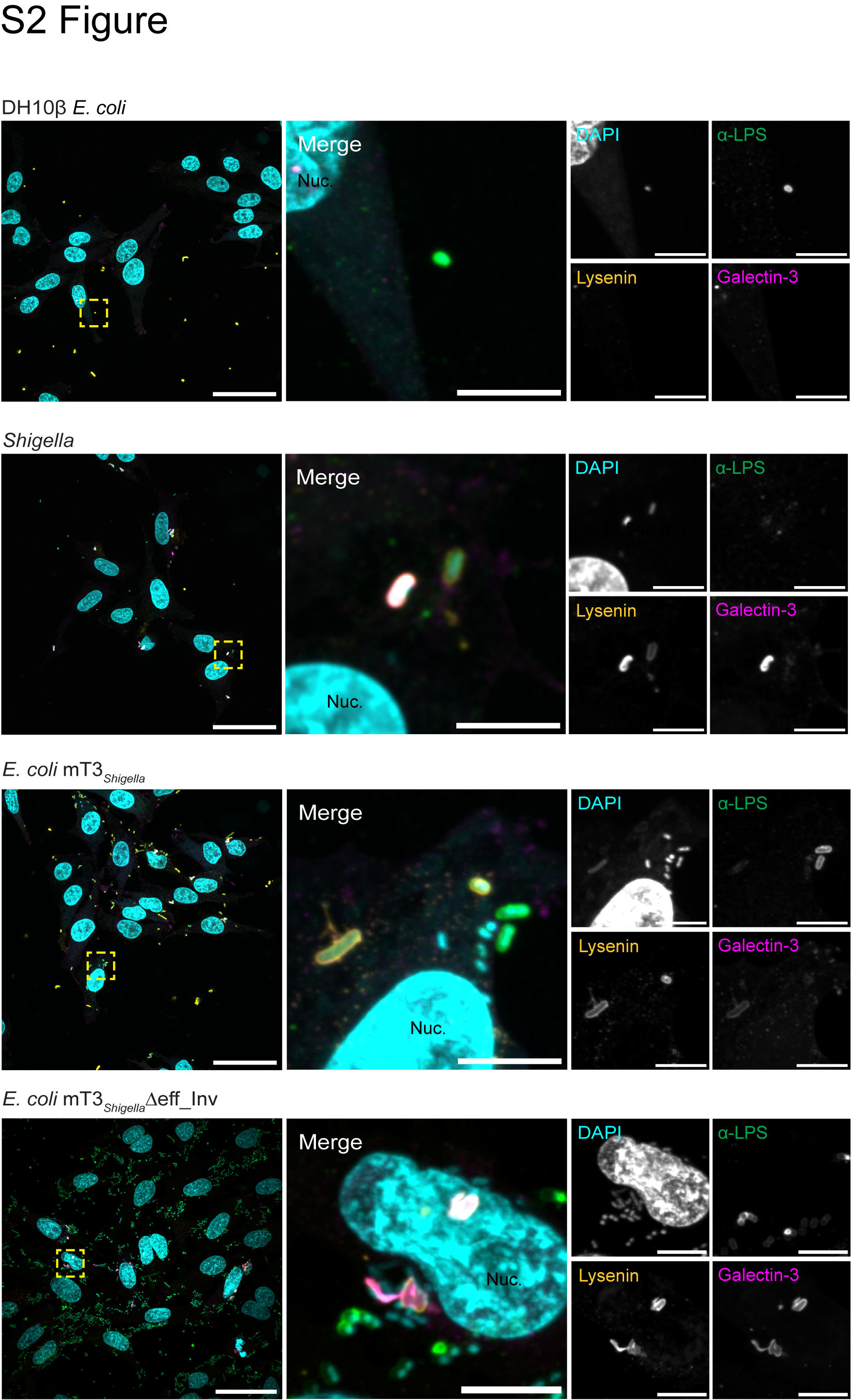

Supplement: S2 Fig — Representative microscopy images of HeLa eGFP-Lysenin mOrange-Galectin-3 cells infected with either DH10β E. coli or E. coli mT3Shigella, for 2 h, E. coli mT3ShigellaΔeff_Inv for 3 h or WT Shigella for 30 min. Extracellular bacteria were stained with an antibody directed against LPS and total bacteria (extra and intracellular) were labeled with DAPI. The First panel shows full field of view, inset is marked by a dashed box. E. coli DH10β did not invade HeLa cells while Shigella, E. coli mT3Shigella and E. coli mT3ShigellaΔeff_Inv did. eGFP-Lysenin: yellow, mOrange-Galectin-3: magenta, DAPI: cyan, and α-LPS: green. Intracellular bacteria: cyan, Extracellular bacteria: cyan and green. Scale bars are 50 µm for the large field of view and 8 µm for all the insets. (TIF) [file pbio.3003135.s002.tif]

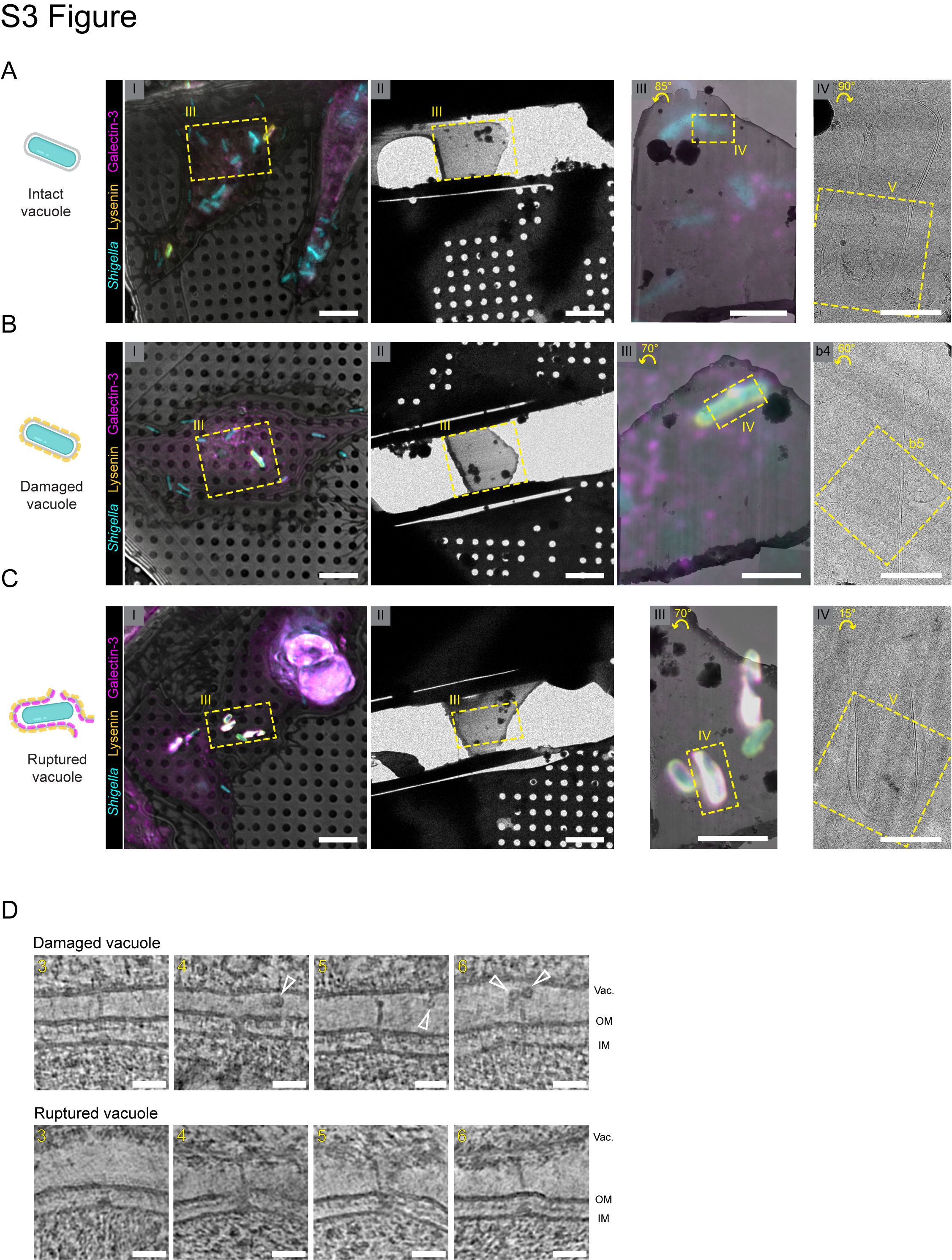

Supplement: S3 Fig — (A–C) Detailed correlation steps for the identification intact (A), damaged (B), or ruptured (C) vacuoles (shown in Fig 2) in HeLa eGFP-Lysenin mOrange-Galectin-3 cells infected with Tag-BFP Shigella processed through a correlative cryo-ET workflow. (I) Vitrified cells on cryo-EM grids were imaged by cryo-fluorescence microscopy to localize infection sites and target them for lamella milling. Tag-BFP Shigella: cyan, eGFP-Lysenin: yellow, and mOrange-Galectin-3: magenta. Scale bars: 10 µm. (II) Cryo-TEM overview of the region targeted in I after cells were thinned into lamellae using cryo-FIB-milling. Scale bars 10 µm. (III) Cryo-lamellae maps overlayed with the corresponding cryo-fluorescence images. Scale bar 5 µm. The rotation angles from images I and II to III are indicated on the top left. (IV) Inset of III showing the bacteria that was targeted for imaging. Scale bar 1 µm. The rotation angles from images III to IV are indicated on the top left. Square boxes V correspond to regions presented in the tomographic slices of Fig 2A, 2B, and 2C respectively. (D) Additional T3SS insets marked in Fig 2 damaged and ruptured panels. Outlined arrowheads point to undetermined densities in the lumen of the Shigella vacuoles. Scale bars are 50 nm. Vac.: Vacuole, OM: Outer membrane, IM: Inner membrane. (TIF) [file pbio.3003135.s003.tif]

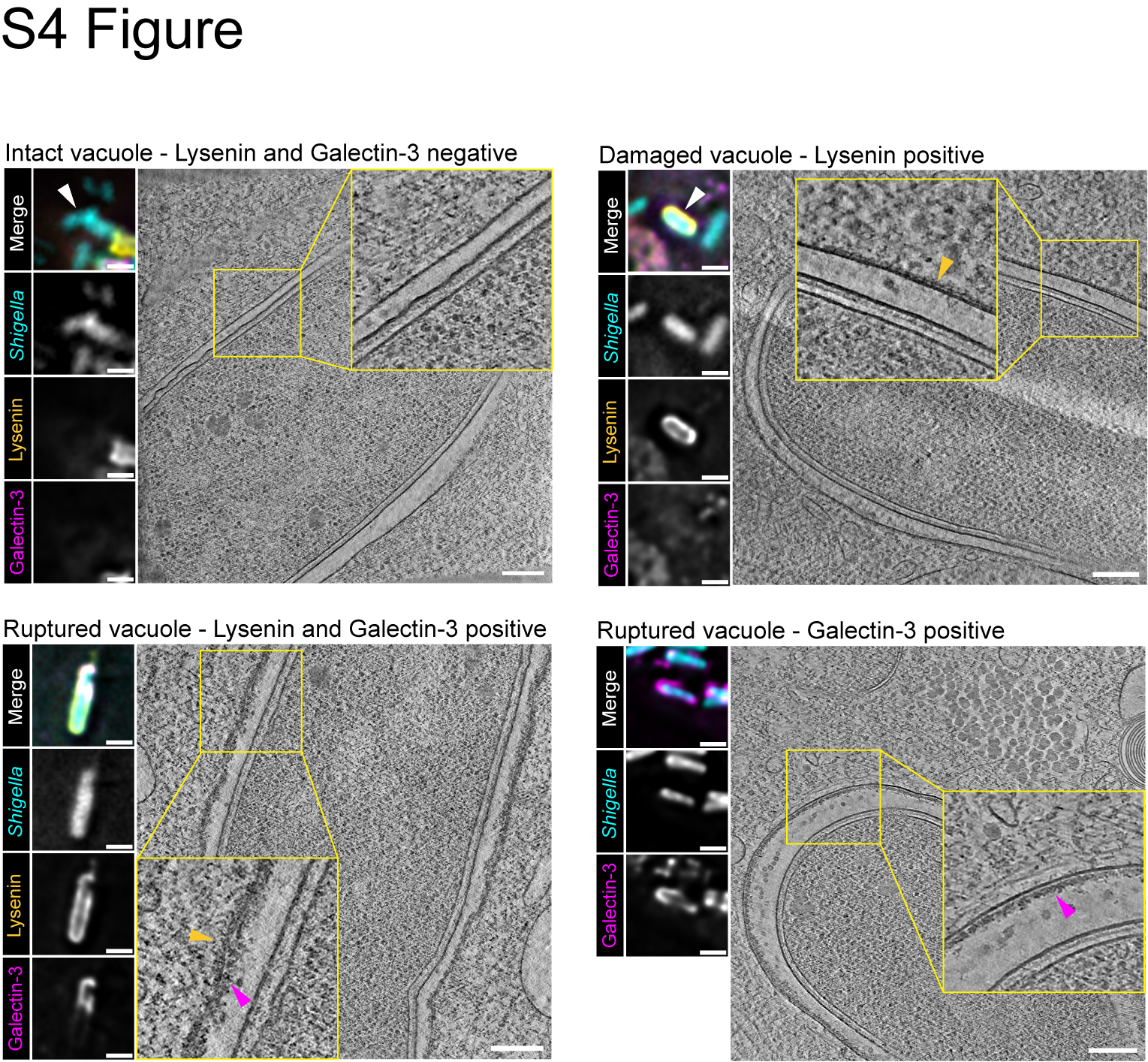

Supplement: S4 Fig — Slice through tomograms (scale bars 200 nm) and corresponding cryo-fLM images (scale bars 2 µm) of Shigella (white arrowhead) at different infection stages with vacuole membranes showing different coating patterns that may reflect on recruitment of overexpressed Lysenin (yellow arrowhead) and Galectin-3 (magenta arrowhead). Intact vacuoles: membrane coating is never observed. Damaged vacuoles: cytosolic side of the vacuole membrane uniformly coated with electron-dense layers upon Lysenin recruitment. Ruptured vacuoles: both on the cytosolic and luminal side of Lysenin and Galectin-3 double-positive vacuoles are coated while only the luminal side of the vacuole membrane is coated in cells expressing just the Galectin-3 marker. (TIF) [file pbio.3003135.s004.tif]

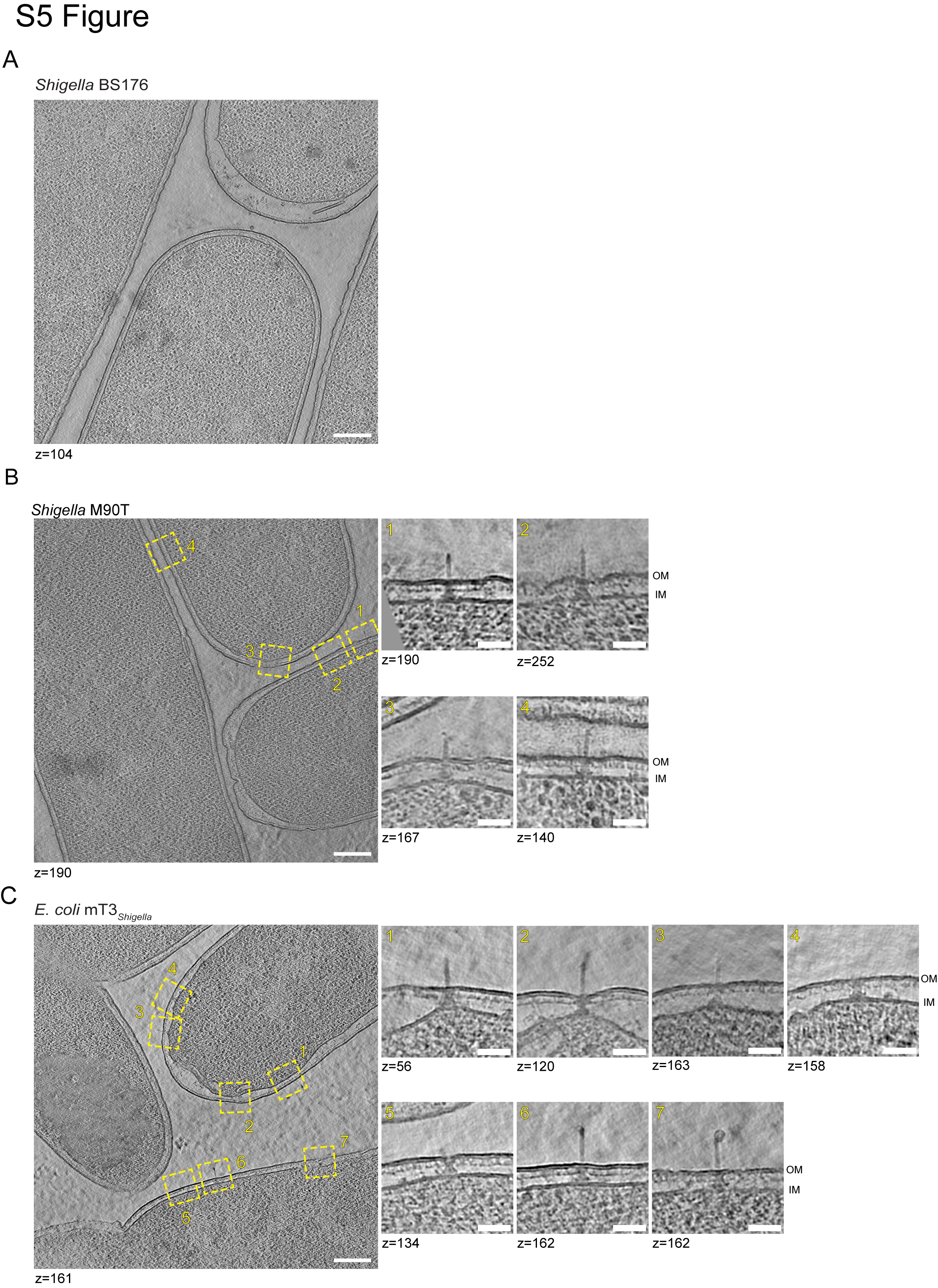

Supplement: S5 Fig — Cryo-tomograms were acquired from cryo-lamellae of various bacterial strains, with T3SS-like complexes identified exclusively in the tomograms of T3SS-encoding strains. Tomographic slices are shown (scale bars 200 nm) and when applicable T3SS regions are marked by a dashed box and corresponding insets shown (scale bars 50 nm). Z slices of tomograms and insets are indicated below the images. Vac.: Vacuole, OM: Outer membrane, IM: Inner membrane. (A) Shigella flexneri BS1276 (pWR100 deficient strain). No T3SSs were seen in tomograms. (B) WT Shigella flexneri M90T carrying the virulence plasmid pWR100, encoding for the T3SS structural component and most of the Shigella secreted effectors. Note that inset n°1 shows a gray area as the T3SS is on the edge of the tomogram. (C) E. coli mT3Shigella, engineered E. coli strain expressing Shigella T3SS structural components including the translocon pore (IpaB, IpaC) and secreted effectors (IpaA, IcsB, IpgD). (TIF) [file pbio.3003135.s005.tif]

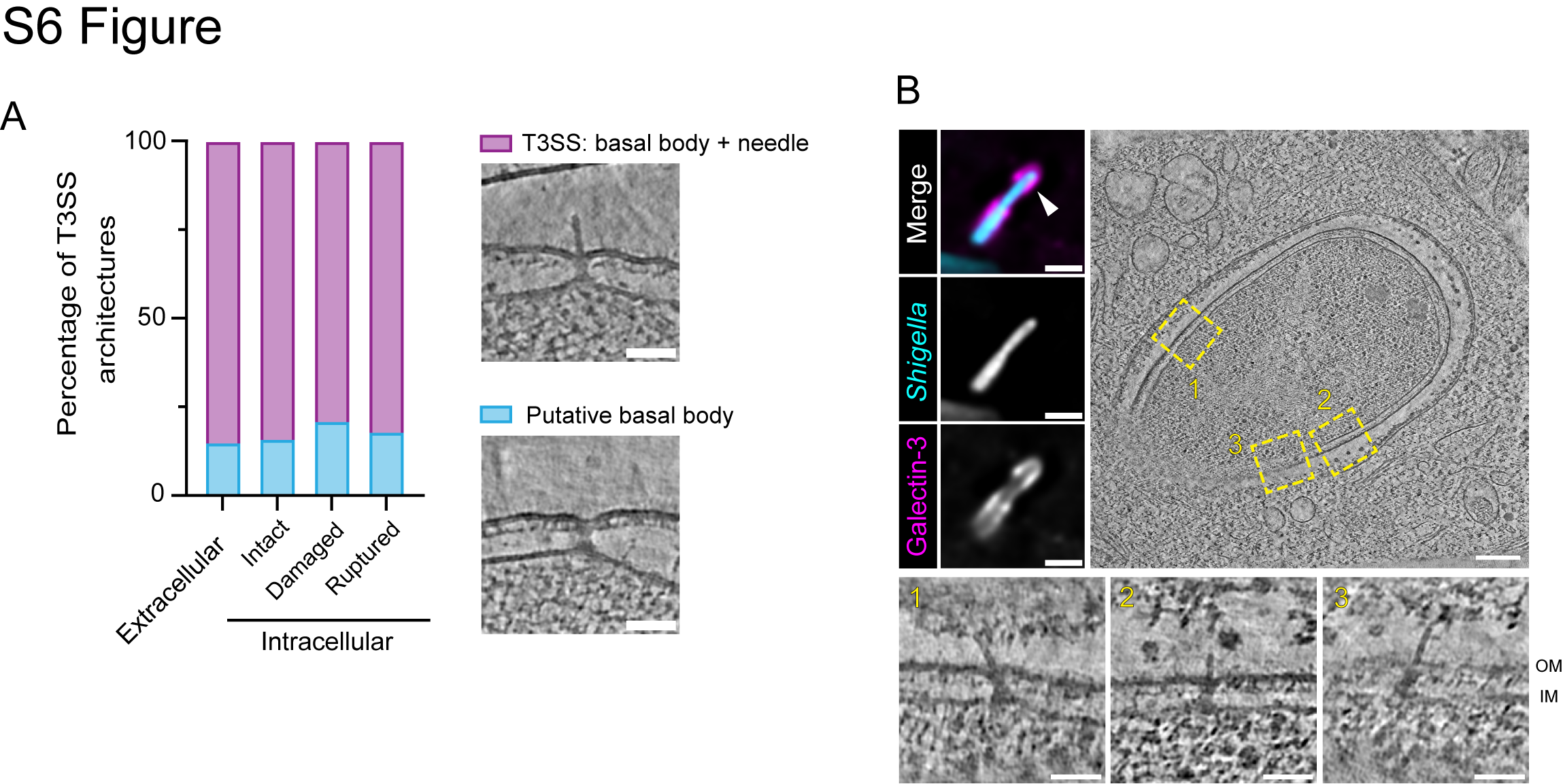

Supplement: S6 Fig — (A) Diversity of T3SS architectures might reflect different assembly states. Contingency graph of the percentage of T3SSs with a membrane-spanning basal body and protruding needle (total n = 122) or with only a basal body without needles (total n = 25) plotted according to the infection stage. Representative examples of putative T3SSs assembly states are shown. Scale bars are 50 nm. The data underlying S6 Fig can be found in S4 Data. (B) T3SS needles are exposed to the cytosol after vacuole rupture and disassembly. Cryo-fLM, (scale bars 2 µm) and corresponding tomogram slice (scale bar 200 nm) of Shigella with ruptured vacuole displaying T3SSs with needles exposed to the cytosol after vacuole rupture (insets, scale bars 50 nm). OM: Outer membrane, IM: Inner membrane. (TIF) [file pbio.3003135.s006.tif]

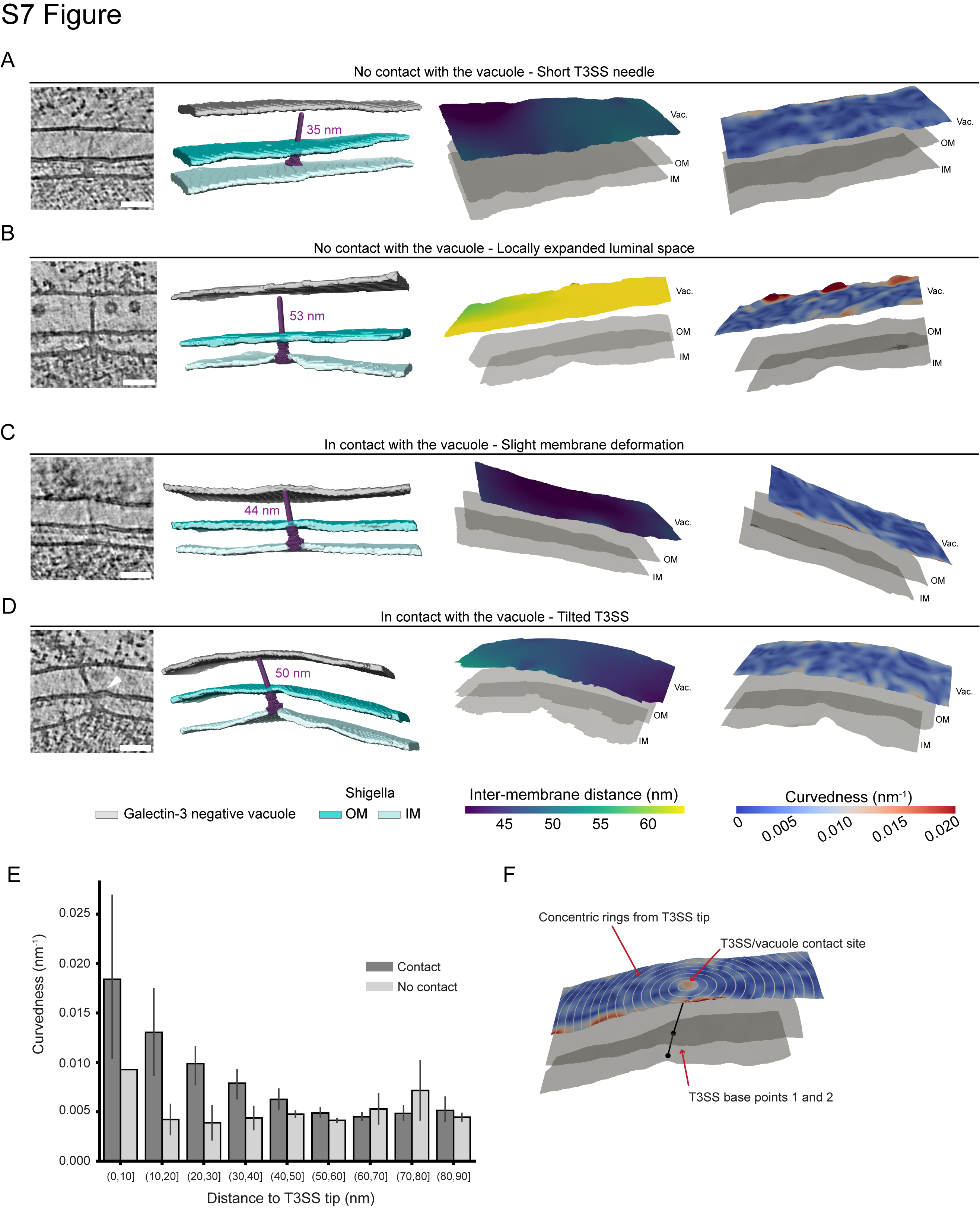

Supplement: S7 Fig — (A–D) Tomogram slices of the T3SS-vacuole zones before Shigella membrane rupture. White arrowhead points to bacterial membranes deformations. Scale bars 50 nm. Corresponding 3D rendering of vacuole and bacteria membranes with Shigella T3SS 3D map (EMD-15700) fitted. Galectin-3 negative vacuole (gray), bacteria OM (dark blue) and IM (light blue). Next panels show membrane surface reconstructions of the vacuole membrane coloured by 3D distance to the bacteria outer membrane (left) or vacuole membrane curvedness (right). Bacteria membranes are shown in light gray. Vac.: Vacuole, OM: Outer membrane, IM: Inner membrane. The data underlying S7 Fig can be found at https://doi.org/10.5281/zenodo.15065516. (A and B) T3SSs do not establish contact with the vacuole if they are short (A) or if the vacuole is locally relaxed (B). (C) T3SS with needle length correlating with the available vacuole space contact the vacuole without deforming it. (D) T3SS with long needle contacting the vacuole membrane adopts tilted insertion within bacterial membranes. (E) Quantification of the vacuole membrane curvedness (nm−1) as a function of the distance from the T3SS needle tip (exemplified in F), depending on whether or not T3SS establishes contact with the vacuole (T3SS tips with a distance smaller than 5 nm were considered to be in contact). Bars show the 5%–95% percentile range. (F) Annotated graphical representation showing curvedness values and their distances to the T3SS needle tip in bins of width 10 nm, as used for the quantification in (E). (TIF) [file pbio.3003135.s007.tif]

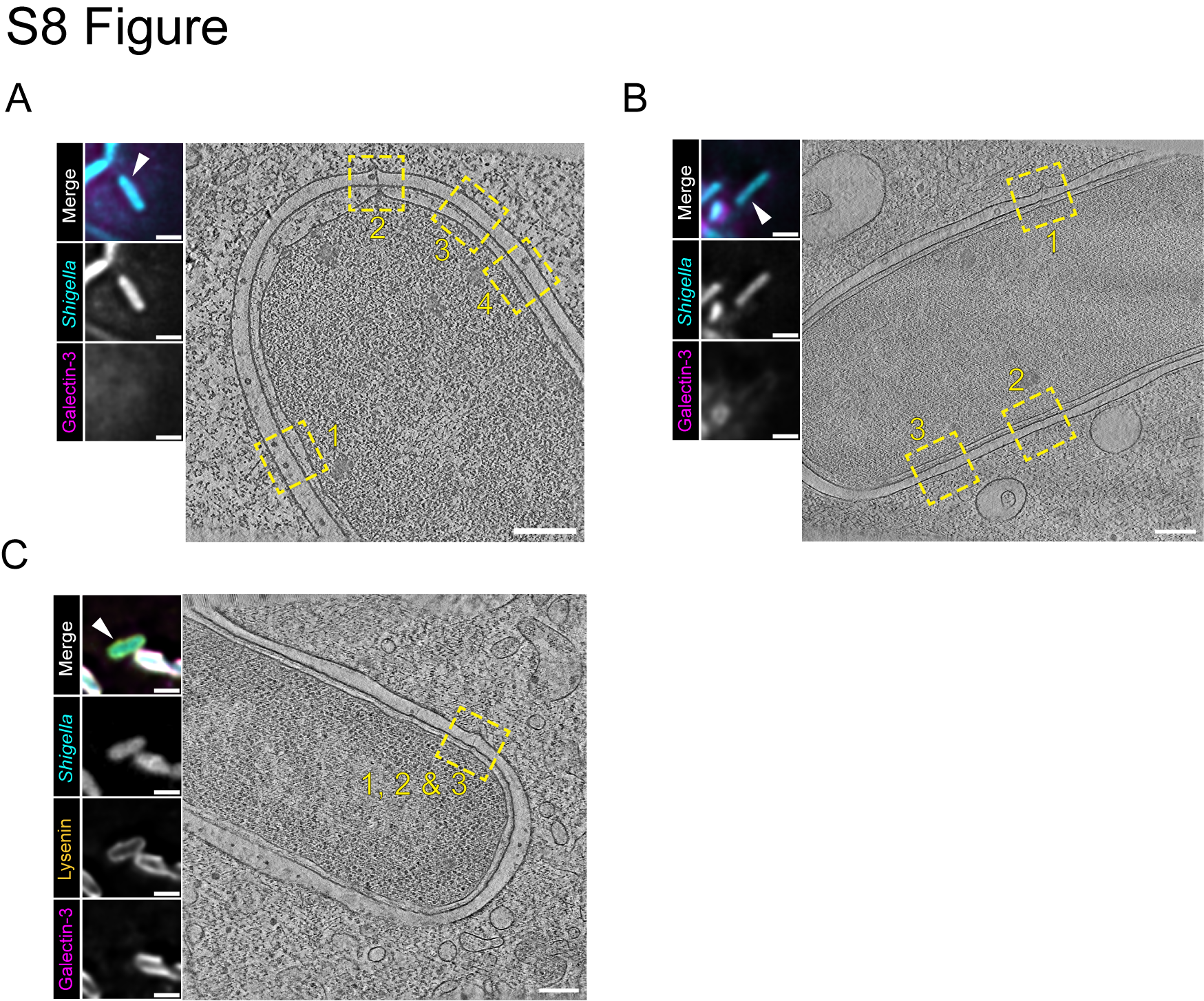

Supplement: S8 Fig — Correlations of the tomogram insets shown in Figs 4 and S7. See also S7–S9 Videos. Slice through tomograms (scale bars 200 nm) and corresponding cryo-fLM images (scale bars 2 µm) of Shigella (white arrowhead) before vacuole rupture. Inset correspondence: (A) T3SS 1: S7B Fig, 2: Fig 4B, 3: S7D Fig, 4: Fig 4A. (B) T3SS 1: Fig 4C, 2: S7D Fig, 3: S7A Fig. (C) T3SSs 1, 2 and 3: Fig 4D. (TIF) [file pbio.3003135.s008.tif]

Full gel - Corresponding to S1 Figure

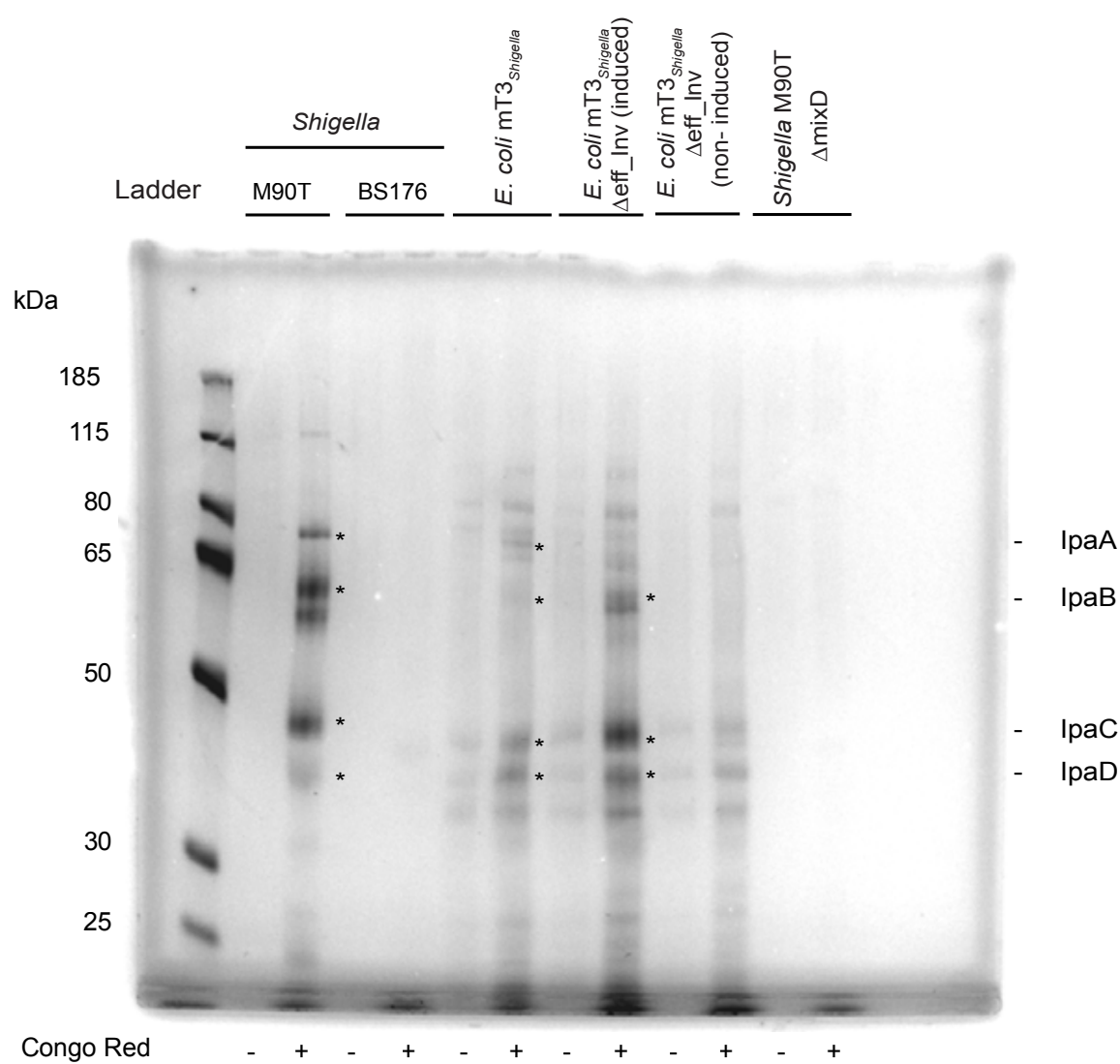

Supplement: S1 Raw Image — (PDF) [file pbio.3003135.s023.pdf]
